# AIM2-Like Receptors Positively and Negatively Regulate the Interferon Response Induced by Cytosolic DNA

**DOI:** 10.1128/mBio.00944-17

**Published:** 2017-07-05

**Authors:** Yuki Nakaya, Jingtao Lilue, Spyridon Stavrou, Eileen A. Moran, Susan R. Ross

**Affiliations:** a Department of Microbiology and Immunology, College of Medicine, University of Illinois at Chicago, Chicago, Illinois, USA; b Department of Microbiology, Perelman School of Medicine, University of Pennsylvania, Philadelphia, Pennsylvania, USA; c Wellcome Trust Sanger Institute, Wellcome Trust Genome Campus, Cambridge, United Kingdom; Brown University

**Keywords:** ALR, Aim2, Trex1, endogenous retrovirus, retrotransposon, self DNA

## Abstract

Cytosolic DNAs derived from retrotransposons serve as pathogen-associated molecular patterns for pattern recognition receptors (PRRs) that stimulate the induction of interferons (IFNs) and other cytokines, leading to autoimmune disease. Cyclic GMP-AMP synthase is one PRR that senses retrotransposon DNA, activating type I IFN responses through the stimulator of IFN genes (STING). Absent in melanoma 2 (AIM2)-like receptors (ALRs) have also been implicated in these pathways. Here we show that the mouse ALR IFI205 senses cytosolic retrotransposon DNA independently of cyclic GMP-AMP production. AIM2 antagonizes IFI205-mediated IFN induction activity by sequestering it from STING. We also found that the complement of genes located in the ALR locus in C57BL/6 and AIM2 knockout mice are different and unique, which has implications for interpretation of the sensing of pathogens in different mouse strains. Our data suggest that members of the ALR family are critical to the host IFN response to endogenous DNA.

## IMPORTANCE

Autoimmune diseases like Aicardi-Goutières syndrome and lupus erythematosus arise when cells of the immune system become activated and attack host cells and tissues. We found that DNA generated by endogenous retroviruses and retroelements in inbred mice and mouse cells is recognized by several host proteins found in macrophages that are members of the ALR family and that these proteins both suppress and activate the pathways leading to the generation of cytokines and IFNs. We also show that there is great genetic diversity between different inbred mouse strains in the ALR genes, which might contribute to differential susceptibility to autoimmunity. Understanding how immune cells become activated is important to the control of disease.

## INTRODUCTION

The innate immune response is initiated when pathogen structures termed pathogen-associated molecular patterns (PAMPs), including DNA, RNA, proteins, and sugar chains, are recognized by pattern recognition receptors (PRRs) that then activate downstream signaling pathways to rapidly induce antipathogen responses (1, 2). When PAMPs generated from endogenous molecules activate these pathways, autoimmune disease can occur. Retrotransposons such as endogenous retroviruses (ERVs) and retrotransposons are believed to be a source of cytosolic DNAs that activate host DNA sensors (3, 4). ERVs, the remnants of ancestral retrovirus infection in the germ line, occupy approximately 10% of the mammalian genome (5). Although most ERVs are inactivated by accumulated mutations and deletions, many are still transcriptionally active and able to produce cytosolic DNAs by reverse transcription (RT) (6). Retrotransposons like line-1s, which are reverse transcribed in the nucleus, also produce cytosolic DNA (4, 7).

Many host molecules have been implicated in the control and recognition of cytosolic DNA generated by either exogenous infection or endogenous retroelements. The cytoplasmic 3′-5′ DNA exonuclease three prime repair exonuclease 1 (TREX1) metabolizes cytosolic DNA to prevent intrinsic DNA accumulation in the cytoplasm (3, 8). Abnormal cytosolic DNA accumulation associated with TREX1 mutations is thought to lead to autoimmune diseases characterized by excessive type I interferon (IFN) and cytokine production, such as Aicardi-Goutières syndrome (AGS), which results in neuronal disorders and death in childhood (3, 9–13). When not degraded by TREX1, cytosolic DNA is recognized by host PRRs that trigger IFN and cytokine production. For example, the sensor cyclic GMP-AMP synthase (cGAS) produces cyclic GMP-AMP (cGAMP) after binding double-stranded DNA (dsDNA); cGAMP then binds to and activates the stimulator of IFN gene (STING) protein (14–17). STING, in turn, activates tank-binding kinase 1 (TBK1) and transcription factor IFN regulatory factor 3 (IRF3), thereby inducing the expression of type I IFN, as well as other cytokines (18). Knockout of *cGas* ameliorates the autoimmune phenotype seen in *Trex1* knockout mice (21, 22).

Absent in melanoma 2 (AIM2)-like receptors (ALRs) have also been implicated in cytosolic DNA recognition. *ALR* genes are found in tandem arrays at the same genomic locus in all mammals except bats (23–29). Interestingly, the ALR locus is highly variable in different species. For example, humans have 4 *ALR* genes, including *AIM2*, while mice have 13 or 14 (26, 29). While the ALRs are believed to be involved in the modulation of type I IFN or inflammasome pathways, the precise roles of most of the individual genes in this locus have not been elucidated (26, 29, 30). AIM2 binding to dsDNA leads to induction of the inflammasome pathway (25, 31, 32). Another human ALR, the IFN-inducible 16 (IFI16) protein, functions as a dsDNA sensor for the induction of type I IFN and inflammasome activation against pathogens (33–38). In previous studies, we showed that reverse-transcribed DNA generated during murine leukemia virus (MLV) infection of macrophages was sensed by cGAS, DEAD box helicase 41 (DDX41), and the mouse ALR IFI203 (39, 40). Thus, multiple DNA sensors may be employed to achieve a type I IFN response.

Our goal here was to determine whether any of the ALRs are involved in the sensing of DNA derived from endogenous retroelements. We show that the mouse ALR IFI205 senses self DNA derived from retrotransposons in the cytoplasm of macrophages and activates the type I IFN signaling pathway via STING. cGAS also sensed self DNA. However, IFI205-mediated activation of the type I IFN response was independent of cGAMP. Interestingly, AIM2 dampened the self DNA-sensing pathway, likely by sequestering IFI205 from STING. These studies are in contrast to a recent publication describing knockout mice lacking the entire *Alr* locus, including *Aim2*, suggesting that ALRs play no role in the recognition of endogenous DNA (27). We suggest instead that ALRs, including IFI205 and AIM2, function together with cGAS as positive and negative regulators of the innate immune response to cytosolic self DNAs.

## RESULTS

### *Aim2* knockdown augments the *Trex1* knockdown-mediated type I IFN response in macrophages.

**FIG 1:**
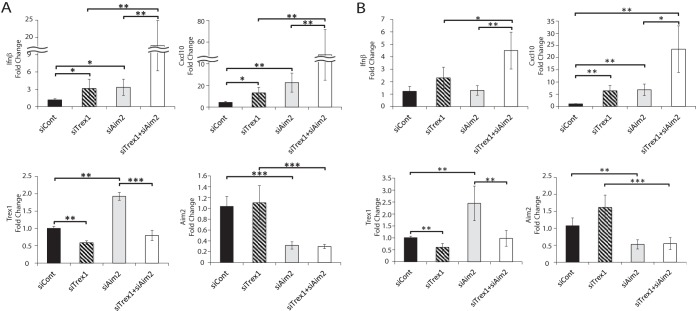
*Trex1* and *Aim2* double knockdown induces a type I IFN response in macrophages. *IFN-β*, *CXCL10*, *Trex1*, and *Aim2* RNA levels upon knockdown of the genes indicated in NR9456 cells (A) and BMDMs (B). RNA were analyzed by RT-qPCR. The values presented are normalized to *Gapdh* and are the mean ± the standard error of the mean of three experiments. *, *P* < 0.05; **, *P* < 0.005; ***, *P* < 0.0005 (two-tailed *t* test). The knockdown of TREX1 and AIM2 protein levels by siRNA treatment is shown in Fig. S2B. siCont, control siRNA.

To determine which molecules are involved in the response to endogenous DNA in macrophages, we carried out a targeted small interfering RNA (siRNA) screening of a panel of genes implicated as cytosolic DNA sensors in the mouse macrophage cell line NR9456; cytosolic sensing in response to exogenous murine retrovirus infection and intact Toll-like receptor pathways in response to arenavirus infection are intact in this cell line (39–41). *Trex1* or *Aim2* knockdown alone resulted in modest but significant increases in levels of RNAs for *IFN-β* and *CXCL10*, a known IFN-stimulated gene (ISG) (Fig. 1A; see Fig. S1A in the supplemental material). The modest induction of *IFN-β* or *CXCL10* RNA levels by *Trex1* knockdown was significantly diminished by *cGas* or *Sting* depletion (Fig. S1). Dual knockdown of *Trex1* and the other potential sensors also resulted in modest or no decreases in cytokine RNA levels (Fig. S1A).

10.1128/mBio.00944-17.1 FIG S1  Initial screening of the molecules involved in the type I IFN response upon Trex1 knockdown. (A) NR9456 cells were transfected with siRNAs as indicated. Changes in the expression levels of the genes indicated were measured by RT-qPCR. Values were normalized to *Gapdh* and are the mean ± the standard error of the mean of six experiments for control siRNA (siCont) and siTrex1+siAim2 and three experiments for the others performed with duplicate technical replicates. (B) Basal gene expression levels were measured by RT-qPCR in NR9456 cells. Values were normalized to *Gapdh* and are the mean ± the standard error of the mean of three different cultures. Download FIG S1, PDF file, 0.3 MB. Copyright © 2017 Nakaya et al. 2017 Nakaya et al. This content is distributed under the terms of the Creative Commons Attribution 4.0 International license.

Interestingly, dual knockdown of *Trex1* and *Aim2* resulted in much higher *IFN-β* and *CXCL10* transcript levels than *Trex1* knockdown alone (Fig. 1A; see Fig. S1A). Similar results were obtained with primary bone marrow-derived macrophages (BMDMs) from BL/6 mice (Fig. 1B). To control for potential off-target effects, we used three different *Aim2* siRNAs; all three caused increased *IFN-β* and *CXCL10* and reduced AIM2 RNA and protein levels (Fig. S2A and B). Since Trex1 is itself an ISG, because of the increase in type I IFN levels, knockdown of Aim2 alone increased Trex1 RNA levels (Fig. 1; see Fig. S2A).

10.1128/mBio.00944-17.2 FIG S2  The effect of siAim2 on the IFN response was not off target. (A) NR9456 cells were transfected with the siRNAs indicated, and expression levels of *IFN*-*β*, *CXCL10*, *Trex1*, and *Aim2* were measured by RT-qPCR. Values were normalized to *Gapdh* and are the mean ± the standard error of the mean of three experiments. *, *P* < 0.05; **, *P* < 0.005; ***, *P* < 0.0005 (two-tailed *t* test). (B) Knockdown effect of each siRNA in macrophages. NR9456 or NR9456-IFI205myc cells were transfected with the siRNAs indicated. TREX1, AIM2, and IFI205myc were detected by Western blotting with the antibodies indicated to the left of each panel. Western blotting for TREX1, IFI205myc, and AIM2 were performed one, one, and two times, respectively. Download FIG S2, PDF file, 0.4 MB. Copyright © 2017 Nakaya et al. 2017 Nakaya et al. This content is distributed under the terms of the Creative Commons Attribution 4.0 International license.

**FIG 2:**
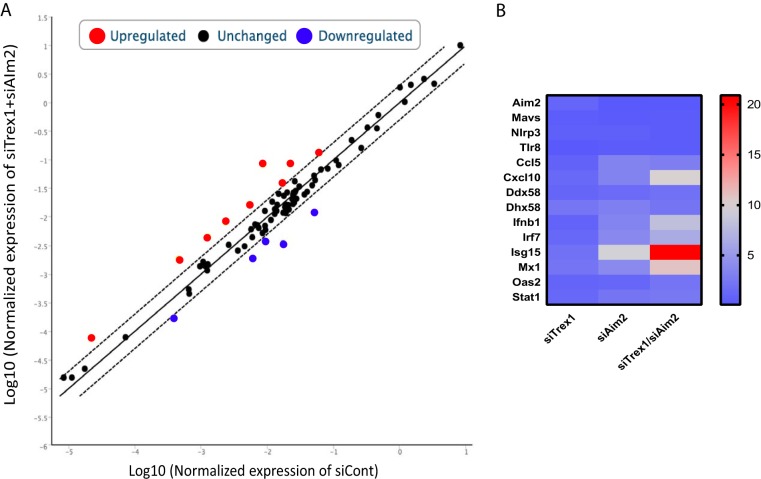
*Aim2* and *Trex1* depletion increases ISG RNA levels. A PCR array of NR9456 cells was conducted after knocking down the genes indicated. (A) Scatterplot of the genes analyzed. (B) Heat map of genes with significant differences from those of control siRNA (siCont)-treated cells. Equal amounts of total RNAs from three different experiments were combined and subjected to cDNA synthesis. A single PCR array was performed.

To further investigate the cellular response to increased cytosolic DNA in AIM2-depleted cells, we performed a PCR array for each knockdown condition (Fig. 2A). Similar to what we found with *IFN-β* and *CXCL10*, several ISGs were modestly induced by *Trex1* or *Aim2* knockdown alone (Fig. 2B; see Fig. S3). However, the expression of ISGs such as *Ccl5*, *Irf7*, *Isg15*, and *Mx1* was greatly increased in response to the knockdown of both genes (Fig. 2B; see Fig. S3). These data suggested that AIM2 was moderating the innate immune response to endogenous cytosolic DNA.

10.1128/mBio.00944-17.3 FIG S3  Expression profiles of individual genes in the PCR array. Downregulated and upregulated genes are blue and red, respectively. Download FIG S3, PDF file, 0.1 MB. Copyright © 2017 Nakaya et al. 2017 Nakaya et al. This content is distributed under the terms of the Creative Commons Attribution 4.0 International license.

### *Trex1* knockdown increases the level of cytosolic retrotransposon DNAs.

**FIG 3:**
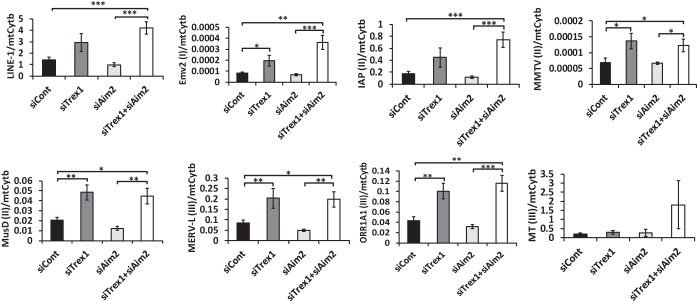
Trex1 knockdown increases cytosolic retrotransposon DNA levels. (A) Cytosolic DNA was isolated from NR9456 cells treated with the siRNAs indicated. The retrotransposon DNA copy number was measured by qPCR and normalized to that of the mitochondrial gene for cytochrome *b* (*mtCytb*). I, II, and III in parentheses indicate class I, II, and III retroviruses, respectively. The values shown are the mean ± the standard error of the mean of three experiments. *, *P* < 0.05; **, *P* < 0.005; ***, *P* < 0.0005 (two-tailed *t* test). siCont, control siRNA.

While a previous study suggested that retroelement DNA contributes to the TREX1-dependent innate immune response, which elements were degraded by Trex1 and whether cytoplasmic versus nuclear DNA was involved was not examined (3). We thus measured the cytosolic DNA levels of retroelements belonging to different families after Trex1 depletion. Cytosolic DNA derived from all endogenous retrotransposons except MT(III) Was upregulated upon Trex1 depletion in the absence or presence of Aim2 knockdown, while total cellular or nuclear retrotransposon DNA did not show consistent increases under any conditions (Fig. 3; see Fig. S4). Aim2 knockdown alone did not affect cytosolic DNA levels of any of the retroelements (Fig. 3). The relative levels of the mitochondrial gene for cytochrome *b* also did not change with any of the knockdowns (Fig. S4).

10.1128/mBio.00944-17.4 FIG S4  Influence of Trex1 and Aim2 knockdown on endogenous retrotransposon copy numbers. NR9456 cells were transfected with siRNAs as indicated. Whole DNA was isolated 72 h after the first transfection to measure the copy numbers of ERVs by qPCR, which were normalized to mtCytb, whose copy numbers did not change upon Trex1 knockdown (final panel). Experiments were performed in triplicate and repeated three times. Download FIG S4, PDF file, 0.2 MB. Copyright © 2017 Nakaya et al. 2017 Nakaya et al. This content is distributed under the terms of the Creative Commons Attribution 4.0 International license.

Raltegravir, which blocks the integration of retroviruses and retroelements, leading to the accumulation of unintegrated nuclear DNA, has been shown to exacerbate autoimmunity in mice genetically predisposed to this condition and to inhibit ERV retrotransposition *in vitro* (42, 43). To determine whether there was nuclear sensing of retroelements, we treated cells with raltegravir at 1 and 10 μM, concentrations 5- and 50-fold higher than those needed to block the integration of MLV viral dsDNA into chromosomes and to inhibit ERV integrases, respectively (43). Raltegravir treatment did not significantly increase *IFN-β* levels (Fig. S5).

10.1128/mBio.00944-17.5 FIG S5  Raltegravir does not increase the type I IFN response in macrophages. NR9456 cells treated with raltegravir at different concentration (0, 0.5, and 5 mg/ml) were transfected with the siRNAs indicated. *IFN-β*, *CXCL10*, *Trex1*, and *Aim2* expression levels were measured by RT-qPCR. Values were normalized to *Gapdh* and are the mean ± the standard error of the mean of three experiments in triplicate. *, *P* < 0.05; ***, *P* < 0.0005 (two-tailed *t* test). Download FIG S5, PDF file, 0.2 MB. Copyright © 2017 Nakaya et al. 2017 Nakaya et al. This content is distributed under the terms of the Creative Commons Attribution 4.0 International license.

These data suggest that the IFN response was caused by the accumulation of cytosolic retrotransposon DNAs that occurs in the absence of TREX1 and the disruption of AIM2 suppression of cytosolic sensing.

### IFI205 is required for increased signaling triggered by AIM2 suppression.

cGAS, ALR family members, and DDX41 have all been implicated in the TREX1/STING-dependent IFN induction pathway (39). To determine which sensors are involved in the AIM2/TREX1-dependent activation of the pathway by endogenous retroelement DNA, we carried out an additional targeted screening with siRNAs targeting *Trex1*, *Aim2*, and potential sensors. *PyhinB* was not tested because we previously showed that it is not expressed in NR9456 cells (39).

**FIG 4:**
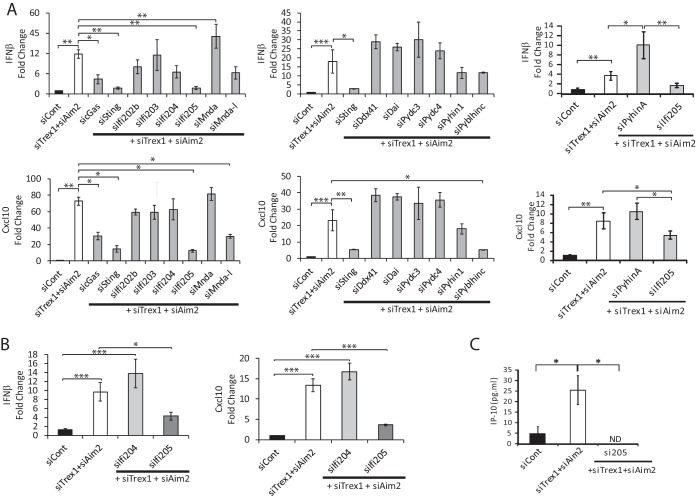
Screening of the genes involved in the type I IFN response. RNAs isolated from NR9456 cells (A) and BMDMs (B) were treated with the siRNAs indicated and analyzed by RT-qPCR with *IFN-β* and *CXCL10* primers. Values were normalized to *Gapdh* and are the mean ± the standard error of the mean of three experiments. Knockdown of each gene was confirmed (Fig. S6). Depletion of IFI205 was also confirmed at the protein level (Fig. S2B). (C) Supernatants of cells transfected with the siRNAs indicated were filtered and subjected to mouse IP-10 ELISA. The values shown are the mean ± the standard error of the mean of three independent experiments. ND, not detectable. *, *P* < 0.05; *, *P* < 0.05; **, *P* < 0.005; ***, *P* < 0.0005 (two-tailed *t* test). siCont, control siRNA.

*Sting* and *cGas* knockdown downregulated the induction of *IFN-β* and *CXCL10* RNAs in TREX1/AIM2-depleted cells (Fig. 4A; see Fig. S6A and B). *Ifi205* depletion also caused a decrease in *IFN-β* and *CXCL10* RNA levels in NR9456 cells (Fig. 4A) and BMDMs (Fig. 4B; see Fig. S6C), comparable to that seen with cGAS or STING depletion, even though its basal level of RNA in NR9456 cells was ~2 orders of magnitude lower than either of these genes (Fig. S1B). *Ifi205* depletion also caused decreased production of CXCL10 (IP-10) protein (Fig. 4C). Knockdown of *Ddx41*, which we showed previously plays a role in the sensing of exogenous retroviral reverse transcripts, did not affect cytokine induction (39). *Pyblhin-c* knockdown decreased *CXCL10* but not IFN-β levels, while treatment of cells with *Mndal* siRNA caused a modest decrease in IFN-β levels and had a greater effect on *CXCL10* levels. In contrast, *PyhinA* knockdown resulted in increases in IFN-β and *CXCL10* RNA levels. Expression of most of the *Alr* genes was increased upon TREX1/AIM2 depletion, consistent with their identification as ISGs (Fig. S6B) (44). Trex1 RNA levels were also higher in Aim2/Trex1 siRNA-treated cells than they were in cells treated with Trex1 siRNA alone although lower than in Aim2 siRNA-treated cells, likely because of the higher IFN levels induced upon double knockdown (compare the relative Trex1 RNA levels in Fig. 1 and Fig. S2 and S6A). However, TREX1 protein levels were similarly depleted in siAIM2- and siAIM2/siTrex1-treated cells (Fig. S2B).

10.1128/mBio.00944-17.6 FIG S6  Gene expression in NR9456 cells. Trex1 (A) and Aim2 (B) target gene expression levels were measured by RT-qPCR with the same cDNAs as in Fig. 4A to examine the knockdown effects of siRNAs. Values were normalized to *Gapdh* and are the mean ± the standard error of the mean of three experiments performed in triplicate. (C) *Trex1* and *Aim2* expression levels were measured with the same BMDM cDNAs as in Fig. 4B to confirm the knockdown effects of the genes. *, *P* < 0.05; ***, *P* < 0.0005 (two-tailed *t* test). Download FIG S6, PDF file, 0.2 MB. Copyright © 2017 Nakaya et al. 2017 Nakaya et al. This content is distributed under the terms of the Creative Commons Attribution 4.0 International license.

**FIG 5:**
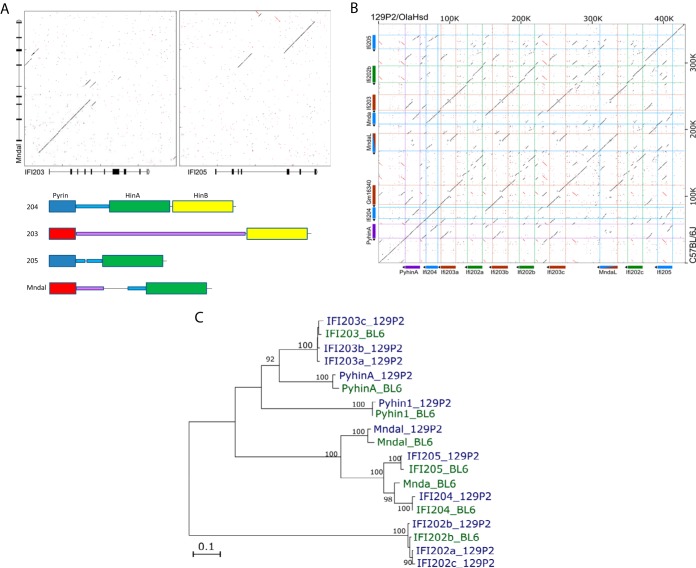
Characterization of mouse *Alr* genes. (A) Dot plot comparing *Mndal* to *Ifi203* from C57BL/6 mice and the predicted domain structures of *Ifi203*, *Ifi204*, *Ifi205*, and *Mndal*. (B) Dot plot comparing the region of the *Alr* locus from *PyhinA* to *Ifi205* in 129P/OlaHsd and C57BL/6J mice. (C) Phylogenetic analysis of ALRs from different mouse strains.

Interestingly, in analyzing the *Alr* locus, we discovered that *Mndal* is a chimeric gene in which the 5′ end is likely derived from *Ifi203* and the 3′ end, containing the Hin DNA binding domain, is derived from *Ifi205* (Fig. 5A). The 3′ Hin domains of *Ifi204*, *Ifi205*, and *Mndal* all belong to the HinA subfamily, while *Ifi203* has a single HinB domain at its C terminus (Fig. 5A) (39). *Ifi203*, *Ifi205* and *Mndal* showed remarkable identity in the noncoding and coding regions (Fig. 5A and B). MNDAL, like IFI205, may also act as a sensor of cytosolic endogenous DNA. However, unlike *Ifi205*, the *Mndal* gene is not present in all mouse strains (data not shown; 45), so its role in sensing was not investigated further.

These results suggested that IFI205 is important for the induction of type I IFN when the endogenous cytosolic DNA level is elevated and AIM2 levels are low.

### The ALR locus differs in 129 and BL/6 mice.

We also attempted to replicate the *Aim2* knockdown studies in BMDMs isolated from *Aim2* knockout mice. However, in analyzing the *Alr* locus in *Aim2* knockout mice, we discovered that while these mice have been extensively backcrossed onto a C57BL/6 background, the region encompassing the *Alr* genes resembled that from the parental 129/P2 embryonic stem cells in which the knockout was created.

To better understand the differences in the BL/6 and 129 *Alr* loci, we first rebuilt the 129P2 *Alr* locus with a bacterial artificial chromosome (BAC) sequence and data from the Sanger Mouse Genomes Project (see Materials and Methods). We further confirmed the assembly by transcriptome sequencing (RNA-Seq) with IFN-β-stimulated 129P2 splenocytes (Fig. S7). While the genome structure of the ALR locus from *Aim2* to *Ifi204* was conserved in BL/6 and 129P2 mice, the region of the locus encompassing *Ifi203*, *Mnda*, *Mndal*, and *Ifi202* differed dramatically (Fig. 5B). Genes in this stretch of the genome showed significant polymorphism and copy number variation. For example, the 129P2 genome contains three copies of diversified *Ifi203* and *Ifi202* genes, compared to the single copy of these genes found in BL/6 mice, and lacks the *Mnda* gene (Fig. 5B). The phylogenetic relationship of these genes is shown in Fig. 5C. Moreover, the expression levels of the different genes throughout the locus differed in the two strains. For instance, *Ifi202b* RNA is barely expressed in BL/6 splenocytes, whereas its homologues are highly expressed in 129P2 splenocytes (Fig. S8A). These studies explain why previous transcription analyses found that not all of the genes in the BL/6 locus were expressed in 129 mice (27); many of the genes examined are not present in the 129 background.

10.1128/mBio.00944-17.7 FIG S7  Illumina reads mapped to the gap between two 129X1 BACs visualized by IGV. From the top to the bottom, 10-kb, 6-kb, and short-insertion Illumina sequences are shown. The sequence from kb 273 to 278 contains transposon elements that cause mismatches and mapping errors. Download FIG S7, PDF file, 0.1 MB. Copyright © 2017 Nakaya et al. 2017 Nakaya et al. This content is distributed under the terms of the Creative Commons Attribution 4.0 International license.

10.1128/mBio.00944-17.8 FIG S8  Comparison of ALRs of C57BL/6 and 129 mouse strains. (A) Profiles of IFN-β-mediated ALR induction in BMDMs from different mouse strains. Shown are the relative read counts from RNA-Seq analysis (see Materials and Methods). (B) Origins of ALRs in Aim2 knockout mice. *, Both copies of IFI202 in 129 mice have a characteristic 400-bp deletion resulting in a smaller PCR product. Download FIG S8, PDF file, 0.1 MB. Copyright © 2017 Nakaya et al. 2017 Nakaya et al. This content is distributed under the terms of the Creative Commons Attribution 4.0 International license.

Using these data, we designed primers to amplify polymorphic regions of genes throughout the region to determine if the entire *Alr* locus in *Aim2* knockout mice was derived from 129 mice. We also used simple sequence length polymorphic (SSLP) primers that flanked the locus. All of the *Aim2* knockout mouse *Alr* genes were derived from the original 129P2 progenitor cells, while other loci, such as the *Apobec3* locus found on chromosome 16, were derived from BL/6 mice (Fig. S8B). This finding is not surprising; given that the distance between the genes for Aim2 and Ifi204 is 0.3 cM (281 kB), it is unlikely that crossover events would have occurred in the seven to eight generations of backcrossing onto C57BL/6J mice. To verify this, we also submitted AIM2 genomic DNA to the mouse universal genotyping array (MUGA) panel and found that the minimum region of chromosome 1 derived from the 129P2 progenitor in *Aim2* knockout mice was from 154868088 to 194886567 (*Aim2* maps to chromosome 1 positions 173350879 to 173466040). Because the *Alr* locus in 129P2 mice was so different from that in C57BL/6 mice, we did not further examine cytosolic DNA sensing in *Aim2* knockout mice.

### AIM2 blocks IFI205 and STING interaction in the cytoplasm.

**FIG 6:**
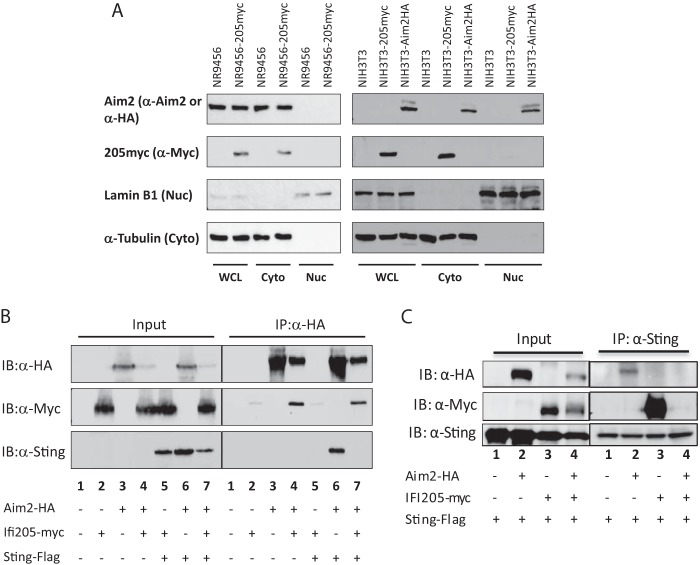
Interaction of IFI205, AIM2, and STING. (A) Intracellular localization of IFI205 and AIM2 in NR9456 and NIH 3T3 cells. Lysates of cells stably expressing IFI205myc or AIM2HA were fractionated into cytoplasmic and nuclear fractions and then subjected to Western blotting with the antibodies indicated. Lamin B1 and α-tubulin were used as markers for the nucleus and cytoplasm, respectively. A single experiment was done for each panel. (B, C) Co-IP of IFI205myc, AIM2HA, and STING-FLAG. HEK293T cells were transfected with tagged proteins as indicated. Cells were lysed 48 h after transfection and immunoprecipitated (IP) with anti-HA (B) or anti-FLAG (C) antibodies. Proteins were detected by Western blotting with the antibodies indicated. The co-IP was repeated three times and gave the same results. WCL, whole-cell lysate; Cyto, cytoplasm; Nuc, nucleus; IB, immunoblotting.

A previous study using a HA-tagged construct transiently transfected into HeLa cells suggested that IFI205 is in the nucleus (26). Because we found that the levels of cytoplasmic but not total endogenous retroelement DNA increased upon Trex1 knockdown and that treatment of cells with raltegravir had no effect on IFN levels, we suspected that sensing by IFI205 occurred in the cytoplasm. First, we determined IFI205’s localization in macrophages and NIH 3T3 cells by cell fractionation. Because antibodies specific for IFI205 are not available, we established NR9456 and NIH 3T3 cells stably expressing myc-tagged IFI205. Interestingly, IFI205 was localized in the cytoplasmic fraction of both macrophages and NIH 3T3 cells (Fig. 6A). We also examined the localization of endogenous AIM2 in macrophages and a hemagglutinin (HA)-tagged version in transfected NIH 3T3 cells (Fig. 6A). Consistent with previous reports, AIM2 was found in the cytoplasm in both cell types; the HA-tagged, but not the endogenous, protein was also found in the nuclei of NIH 3T3 cells (Fig. 6A) (26).

Next, we performed cotransfection/coimmunoprecipitation (co-IP) assays to determine whether IFI205 and AIM2 interact with each other or with STING (Fig. 6B). We coexpressed IFI205myc, AIM2HA, and FLAG-tagged STING in HEK293T cells, immunoprecipitated them with either anti-HA (AIM2) or anti-FLAG (STING) antiserum, and then probed Western blots with antibodies against the three tags; we were unable to immunoprecipitate them with anti-myc antiserum because of nonspecific binding of proteins that interfered with detection by Western blotting (data not shown). Immunoprecipitation with anti-HA antiserum demonstrated that IFI205 and AIM bound each other in the absence (Fig. 6B, lane 4) or presence (Fig. 6B, lane 7) of STING. IFI205 also bound to STING (Fig. 6C, lane 3). However, IFI205/Sting binding was abrogated in the presence of Aim2 (Fig. 6C, lane 4). AIM2 also bound to STING only in the absence of IFI205 (Fig. 6B, lane 7, and C, lane 3).

**FIG 7:**
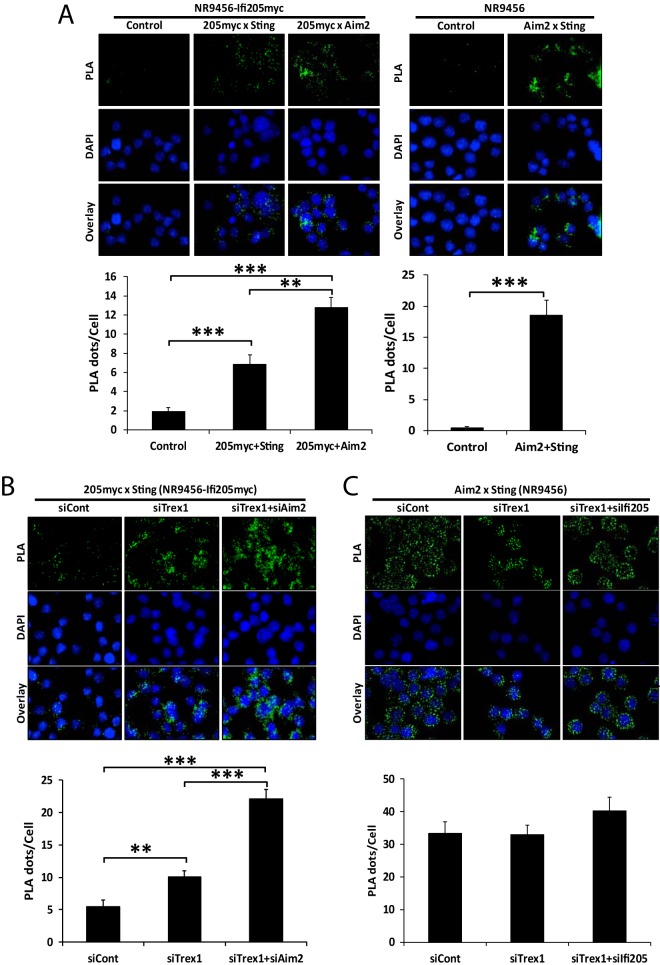
IFI205-STING interaction increases upon TREX1 and AIM2 depletion. (A) PLA for interactions of IFI205myc-AIM2, IFI205myc-STING, and AIM2-STING in NR9456-IFI205myc or NR9456 cells. The number of cells and images quantified for each condition in NR9456-IFI205myc cells are as follows: IFI205myc-Sting, 141 cells and 10 images; IFI205myc-Aim2, 101 cells and 6 images; control, 65 cells and 5 images. The number of cells and images quantified for each condition in NR9456 cells are as follows: control, 74 cells and 5 images; Aim2-Sting, 47 cells and 5 images. Controls for knockdowns are shown in Fig. S9A. (B) PLA for IFI205myc-STING interactions in NR9456-IFI205myc cells with knockdown of the genes indicated. Quantification: siCont, 102 cells and 5 images; siTrex1, 190 cells and 7 images; siTrex1+siAim2, 108 cells and 5 images. A representative image is shown. This experiment was repeated twice and gave similar results both times. (C) PLA for AIM2-STING interaction in NR9456 cells after knockdown of the genes indicated. Quantification: siCont, 84 cells and 4 images; siTrex1, 33 cells and 4 images; siTrex1+si205, 58 cells and 4 images. PLA dots were quantified and normalized to cell numbers based on DAPI staining with ImageJ software. The values shown are the mean ± the standard error of the mean of different pictures. **, *P* < 0.005; ***, *P* < 0.0005. (two-tailed *t* test). siCont, control siRNA.

To further explore the interaction of the three proteins, we also conducted proximity ligation assays (PLA) in which the binding of two proteins to each other produces signals that appear as fluorescent dots. NR9456-IFI205myc and NR9456 cells were used to see the interactions between IFI205 and Aim2, IFI205 and STING, and AIM2 and STING. As in the co-IPs, IFI205 interacted with both AIM2 and STING (Fig. 7A, left side). Endogenously expressed AIM2 and STING also interacted with each other (Fig. 7A, right side). All of the interactions occurred in the cytoplasm (Fig. 7A).

10.1128/mBio.00944-17.9 FIG S9  Knockdown effects of siRNAs in PLA. (A) NR9456-IFI205myc cells were transfected with the siRNAs indicated to confirm reduction of the IFI205myc-STING or AIM2-STING interaction. PLA dots were counted and normalized to cell numbers based on DAPI staining. The values shown are the mean ± the standard error of the mean of different pictures. Cell (picture) numbers: 110 (5) and 156 (5) for siCont and siIfi205 of IFI205myc-STING, respectively; 92 (4) and 111 (4) for siCont and siAim2 of Aim2-STING, respectively; 103 (4), 96 (4), and 69 (4) for siCont, siTrex1, and siTrex1+siIfi205 of Aim2-STING, respectively. *, *P* < 0.05; **, *P* < 0.005 (two-tailed *t* test). (B) Levels of *Ifi205myc* and *Sting* expression measured by RT-qPCR upon knockdown of the genes indicated. Values were normalized to *Gapdh* and are the mean ± the standard error of the mean of three experiments. Download FIG S9, PDF file, 0.7 MB. Copyright © 2017 Nakaya et al. 2017 Nakaya et al. This content is distributed under the terms of the Creative Commons Attribution 4.0 International license.

We also investigated whether depletion of *Trex1*, *Ifi205*, or *Aim2* alters these interactions *in situ*. TREX1 depletion increased the interaction between IFI205 and STING, showing that this interaction is enhanced by IFI205 binding to DNA (Fig. 7B). The IFI205-STING interaction was increased to an even greater extent when both AIM2 and TREX1 were depleted, again demonstrating that AIM2 blocks the interaction of IFI205 with STING (Fig. 7B). The interaction between AIM2 and STING was not changed, regardless of the presence or absence of TREX1 or IFI205 (Fig. 7C; see Fig. S9A). The conflicting results of the co-IP and PLA studies might be due to the different amounts of IFI205 used in the two experiments; *Ifi205* is expressed at much lower levels than *Aim2* in NR9456 cells (Fig. S1B), while the expression level was comparable to that of AIM2 in the co-IP experiments done by transient transfection into 293T cells (Fig. 6B). The higher levels of IFI205 in the co-IP experiments may have allowed it to compete more effectively with AIM2 for binding to STING. Since *Ifi205* in these cells is under the control of the cytomegalovirus promoter, knockdown of *Trex1* did not result in type I IFN-mediated increases in *Ifi205* transcripts (Fig. S9B).

Taken together, these results indicated that AIM2 blocked the interaction between IFI205 and STING IFI205 and that AIM2 and STING interacted with each other to modulate the type I IFN signals in macrophages.

### IFI205 and cGAS bind retroelement DNA.

**FIG 8:**
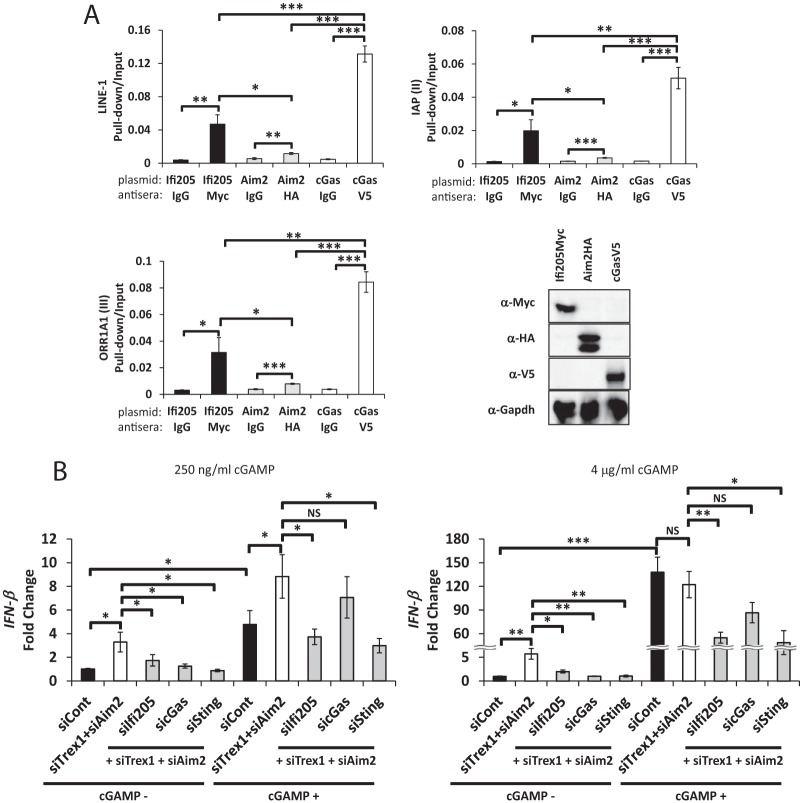
IFI205 binds to the cytoplasmic DNAs of endogenous retrotransposons and induces a cGAMP-independent IFN response (A) DNA pulldown assay for IFI205, AIM2, and cGAS. NIH 3T3 cells were transiently transfected with expression plasmids for IFI205myc, AIM2HA, or cGASV5 and harvested 48 h after transfection. The cytoplasmic fractions were subjected to a DNA pulldown assay with the antibodies indicated. Normal IgG was used as a negative control. Bound DNA copy numbers were measured by qPCR and normalized to input DNA values. The assays were repeated three times for each protein, and qPCR was performed in triplicate for each experiment. Protein expression was confirmed by Western blotting with the same lysates and antibodies used for the DNA pulldown. (B) NR9456 cells were transfected with the siRNAs indicated and cGAMP at the concentrations shown. *IFN-β* expression levels were measured by RT-qPCR and normalized to *Gapdh*. The values shown are the mean ± the standard error of the mean of three experiments. *, *P* < 0.05; **, *P* < 0.005; ***, *P* < 0.0005; NS, not significant (two-tailed *t* test). siCont, control siRNA.

We next used DNA pulldown assays to determine if IFI205, cGAS, or AIM2 bound directly to cytosolic retroelement DNA. We expressed the tagged proteins in NIH 3T3 cells and carried out immunoprecipitations, followed by elution of the DNA bound to the proteins and quantitative PCR (qPCR) with primers for the different retroelements. cGAS bound to all of the endogenous retrotransposon DNAs examined (Fig. 8A) (39). IFI205 bound to the DNAs as well, although the amounts were slightly smaller than those seen with cGAS (Fig. 8A). Notably, AIM2, which is known to bind and sense pathogen DNA (31, 47), bound to the retrotransposon DNA but at levels much lower than those seen with IFI205 and cGAS. Because the expression levels of the three proteins in NIH 3T3 cells were similar (Fig. 8A), AIM2 binding affinity for endogenous DNA may be lower than that of cGAS or IFI205. These results suggest that IFI205 and cGAS are cytosolic DNA sensors that bind to intrinsic cytosolic DNA and STING to activate the type I IFN signal.

### IFI205 sensing of cytosolic self DNA does not depend on cGAMP.

We next tested whether IFI205 functions in the same pathway as cGAS or independently. We treated NR9456 cells with either 200 ng/ml or 4 μg/ml cGAMP, the STING-binding ligand generated when cGAS binds DNA, in conjunction with TREX1, AIM2, and IFI205, cGAS, or STING depletion (Fig. 8B). cGAMP treatment at both concentrations induced the IFN-β response via STING (Fig. 8B). As expected, cGAS knockdown did not significantly affect the cGAMP-mediated increase in IFN-β levels. In contrast, *Ifi205* knockdown reduced IFN-β induction in the presence of both levels of exogenous cGAMP to levels similar to those seen with *Sting* knockdown (Fig. 8B). This indicates that IFI205 binding to cytosolic endogenous DNA induces a type I IFN response independent of cGAMP production.

## DISCUSSION

Autoimmune diseases like AGS are clearly linked to nucleic acid metabolism since mutations in *TREX1*, *RNASEH2A-2C*, *SAMHD1*, *ADAR1*, or *IFIH1* predispose individuals to disease (12, 48–51). Cytoplasmic DNA derived from retroelements are likely responsible, at least in mice, for generation of the ligands that activate nucleic acid sensor-driven pathways. TREX1, which degrades cytosolic DNA, inhibits retrotransposition *in vitro*, and *Trex1* knockout mice develop autoimmune myocarditis that is ameliorated by reverse transcriptase inhibitors (3, 52). Here we show that cytosolic retrotransposon DNAs were increased by TREX1 depletion and that Aim2, Ifi205, and cGAS participated in both positive and negative regulation of the IFN/cytokine response to these DNAs.

Our findings suggest that AIM2 suppresses a signaling pathway that involves the binding of IFI205 and cGAS to cytoplasmic endogenous DNA. IFI205 was previously identified as a candidate cytosolic dsDNA sensor by proteomic screening for ISGs (53). IFI205 is also an inducer of adipogenic differentiation and localizes in the nuclei of osteosarcoma and adipose-tissue-derived stem cells, where it interacts with several transcription factors (54, 55). We showed, however, that IFI205 is localized in the cytoplasm in fibroblasts and in macrophages, the latter of which is a cell type that is probably important for the sensing of both endogenous and exogenous cytosolic DNA. This difference in localization between our studies and those of others may be due to cell type differences, different molecular tags (HA versus myc), or transient versus stable transfection. Moreover, the anti-IFI205 peptide antibodies used in one study would also recognize MNDA and MNDAL (54). Our finding of Ifi205’s cytosolic location in macrophages is consistent with its role in the sensing of endogenous reverse transcripts.

Several recent studies have also suggested that AIM2 antagonizes the type I IFN response in macrophages (27, 56, 57). However, the mechanism by which AIM2 antagonizes this signaling was not elucidated. We show that AIM2 bound to both IFI205 and STING, thereby blocking the IFI205-STING interaction. This, in turn, attenuated the induction of type I IFN by the IFI205-STING pathway. ALRs have a pyrin-PAAD-DAPIN (PYD) domain and at least one hematopoietic IFN-inducible nuclear antigen with a 200-amino-acid repeat (HIN) domain (23, 24, 31, 32). Previous work has shown that the ALR HIN domains bind DNA, while the PYD domains are important for protein-protein interactions (25, 31, 32). Future work will determine if the PYD AIM2 and IFI205 domains allow them to interact with each other and with STING.

Our studies, as well as others, suggest that the pathway initiated by DNA sensing by ALRs is distinct from cGAS, although both converge on STING and are suppressed by AIM2 (39, 58). cGAS is a critical versatile sensor that is able to sense dsDNAs derived from a broad range of pathogens, including endogenous retrotransposons (17, 39). In contrast, proteins belonging to the ALR family seem to have more specific targets. For example, our lab previously demonstrated that IFI203, but no other PYHIN proteins encoded by the murine ALR locus, sensed exogenous MLV reverse-transcribed DNA (39). Moreover, several labs have demonstrated that IFI16 is a nuclear sensor for herpes simplex virus 1 (an alphaherpesvirus), but not for human cytomegalovirus (a betaherpesvirus), in human macrophages (27, 36). Here we showed that IFI205 and perhaps MNDAL, but not the other murine ALRs, are responsible for the sensing of endogenous retroelement DNA. It is not clear why multiple sensors that act through the same downstream STING effector molecule (e.g., cGAS and ALRs) are required to sense cytosolic DNAs. This differential sensing of seemingly similar DNAs by various ALRs could be due to the recognition of specific sequences or PAMP structures or to differential subcellular localization of the nucleic acids generated by exogenous virus infection versus endogenous reverse transcripts. It may also be that there are tissue-specific differences in the expression of the different *Alr* genes that would affect their role in both pathogen responses and autoimmunity.

A recent study using mice with targeted deletion of *Trex1* and the entire *Alr* locus, including *Aim2*, showed that the type I IFN response to self and foreign DNA was independent of ALRs but completely dependent on cGAS, particularly in BMDMs and embryonic fibroblasts (27). This report also showed that deletion of *cGas*, but not the *Alr* locus, rescues *Trex1* knockout mice from autoimmune cardiopathy. It is possible that the physiology of *Trex1* knockout mice, which produce large amounts of cGAMP, alters the balance between sensors that respond to endogenous DNA (21). It may also be that cGAS-mediated induction of cytokines leading to autoimmunity is dominant in the cells that trigger cardiomyopathy; indeed, it has recently been suggested that *Trex1* knockout in dendritic cells alone is sufficient to induce disease in mice (10). Moreover, while loss of only one allele of cGAS was sufficient to ameliorate autoimmunity/cardiomyopathy in *Trex1* knockout mice, knockout of both cGAS alleles was needed to prevent polyarthritis in DNase III knockout mice (21).

Although we found that cytoplasmic and not nuclear DNA activated the AIM2/IFI205 sensing pathway, a previous study showed that the integrase inhibitor raltegravir exacerbated the autoimmune phenotype in two different autoimmune models: (NZB × NZW)F1 mice, which are predisposed to glomerulonephritis, and NZB mice, which are predisposed to autoimmune hemolytic anemia (42). Previous studies have also mapped lupus erythematosus susceptibility genes to the ALR locus (59). We showed here that there are large differences in the complement of ALR genes found in BL6 and 129 mice. We have begun to examine the ALR locus in different inbred mouse strains and found high variability among several *Alr* genes in different strains (unpublished data). As we show here for *Aim2* knockout mice, it will be important to characterize the locus in different mouse strains when determining genetic susceptibility to pathogen infection, inflammation, and autoimmunity. For example, there may be *Alr* genes in the NZB or NZW genetic background that encode variant sensors found in the nucleus that predispose them to exaggerated responses to increased nuclear retroelement DNA induced by raltegravir. Future work to precisely define the complement of ALR genes in different mouse strains will aid in understanding the complex genetics of autoimmunity. However, gene-specific knockout in this region will be difficult to achieve because of the high level of identity in the coding and noncoding regions; this is especially true for *Ifi203*, *Ifi205*, and *Mndal* (Fig. 5A and B).

Given the diversity of ALRs among species or strains, ALRs might have evolved under positive selection by different selective pressures, such as retrotransposons, exogenous viruses, and bacteria, and each *Alr* gene could have different targets or function in different cell types, for example, cells other than macrophages. It is will be important to dissect the roles of individual ALRs to determine how they function in the innate immune network system.

## MATERIALS AND METHODS

### Mice.

C57BL/6N (Charles River, Inc.), 129P2/OlaHsd (Harlan), and B6.129P2-*Aim2*^Gt(*CSG445*)*Byg*^/J knockout (Jackson Laboratory) mice were housed in the animal facilities at the University of Pennsylvania or the University of Illinois at Chicago. All procedures were carried out in accordance with the guidelines approved by the Institutional Animal Care and Use Committee of the University of Pennsylvania and the Animal Care Committee of the University of Illinois.

### Isolation of BMDMs.

Macrophage progenitors were isolated from the femurs of mice and cultured in medium containing 10 ng/ml macrophage colony-stimulating factor (M-CSF) for 6 days to allow them to differentiate into macrophages (60).

### Cells.

HEK293T and NIH 3T3 cells were cultured in Dulbecco’s modified Eagle’s medium (DMEM) supplemented with 10% fetal calf serum, 100 IU/ml penicillin, and 100 μg/ml streptomycin. BMDMs and NR9456 cells, a C57BL/6 mouse macrophage cell line, were cultured in the same DMEM supplemented with 0.1% sodium pyruvate. BMDMs were maintained with 10 ng/ml M-CSF. All cells were maintained at 37°C in a humidified atmosphere of 5% CO_2_ in air. The cell lines have not been tested for mycoplasma contamination within the past year.

### siRNA-mediated knockdown.

The siRNAs used in knockdown experiments are listed in Table S1. NR9456 cells and BMDMs were cultured in 96-well plates for 1 day prior to transfection. NR9456 cells were transfected with either siRNA for Trex1 (siTrex1) or control siRNA (siCont) on day 1 with Lipofectamine RNAiMAX (Invitrogen) and then subjected to secondary transfection with siTrex1 and gene-specific siRNAs on day 2. Cells were harvested on day 4 for RNA isolation. BMDMs were transfected with siTrex1 and gene-specific siRNAs on day 1, transfected with the same siRNAs again on day 3, and harvested on day 5. Total RNA was isolated with the RNeasy kit (Qiagen), and cDNA synthesis was performed with SuperScriptIII and random hexamers (Invitrogen). qPCR was performed with Power SYBR green PCR master mix (Applied Biosystems). The primer sets used are described in Table S1.

10.1128/mBio.00944-17.10 TABLE S1  Real-time PCR primers (a) and siRNA sequences (b) used in this study. Download TABLE S1, DOCX file, 0.01 MB. Copyright © 2017 Nakaya et al. 2017 Nakaya et al. This content is distributed under the terms of the Creative Commons Attribution 4.0 International license.

### PCR array.

NR9456 cells were transfected with Trex1, Aim2, or Trex1 and Aim2 siRNAs. cDNAs from the transfected cells were analyzed by RT^2^ Profiler PCR Mouse antiviral Response Array (Qiagen) in 384-well plates. The data were analyzed by RT^2^ Profiler PCR Array Data Analysis version 3.5 (Qiagen).

### ELISAs.

Supernatants from cells were harvested, and IP-10 was measured with the mouse IP-10 SimpleStep enzyme-linked immunosorbent assay (ELISA) kit (Abcam, Inc.).

### RNA-Seq.

BMDMs were isolated from the femurs of 2-month-old C57BL/6N and 129P2/Ola mice and cultured as described above. After 6 days, they were induced with 2,000 U of IFN-β (PBL Assay Science) for 8 h. RNA was isolated with Trizol (Invitrogen), followed by RNA cleanup with RNeasy kits (Qiagen). RNA-Seq libraries where prepared from approximately 320 ng of total RNA (RNA integrity number between 9 and 10 by Agilent BioAnalyzer) with the Illumina Stranded mRNA library prep kit. The resulting RNA-Seq libraries where sequenced on an Illumina NextSeq 500 in 125-bp paired-end mode (RTA version 2.4.6), generating 30,000 million base-pair reads clean data per sample. FASTQ files where generated with bcl2fastq version v2.15.0.4 and then mapped to the GRCm38 reference sequence with STAR aligner v 2.4.1c (61). For mouse strain 129P2/Ola, all reads mapped to the ALR locus (173.42 to 174.04 million base-pairs the reference sequence) were further extracted with SAMtools v 1.3 and their corresponding read pair mates (either mapped or unmapped). This collection of reads was further mapped with STAR aligner to the reassembled ALR contig of strain 129P2. The expression level of each gene was evaluated by the average read coverage on each exon.

### Reassembly of the ALR locus of 129P2/OlaHsd.

For the conserved region, 129P2/OlaHsd whole-genome Illumina reads mapped to the reference genome were extracted from released BAM files of the Sanger Mouse Genomes Project (62) (ftp://ftp-mouse.sanger.ac.uk/REL-1210-BAM/129P2_OlaHsd.bam) with SAMtools v 1.3 and then remapped to the corresponding region of the 129S1/SvlmJ *de novo* assembly (ftp://ftp-mouse.sanger.ac.uk/REL-1509-Assembly/129S1_SvImJ.chromosomes.unplaced.gt2k.fa.gz) with Geneious R7. Gaps were manually filled by the Illumina short-read and 3-, 6-, and 10-kb long-insertion library. The polymorphic region of the ALR locus was reconstructed with a BAC shotgun sequence from mouse strain 129X1/SvJ (63) (GenBank accession numbers NT_187017 and NT_039195), and verified by whole-genome Illumina reads from 129P2/OlaHsd. The two 129 substrains were confirmed to have the same haplotype at the ALR locus. Judging by the long-insertion Illumina library data, a 26.4-kb gap between NT_187017 and NT_039195 was filled by whole-genome Illumina reads. Details of the reassembly are shown in Fig. 6B; the mapped BAM files are available on request. The reassembled contig was then annotated by AUGUSTUS (64). Dot plot figures were generated with software LBDOT (68).

### Genotyping.

Tail DNA or splenic RNA was analyzed by PCR with primers specific for *PyhinA*, *Pydc3*, *Ifi202*, and *Apobec3*, as well as SSLP primers D1MIT113, D1MIT205, and D1MIT150 (65). The sequences of the primers used are in Table S1. The primers used for *Apobec3* were previously described (66). DNA from C57BL/6N, 129P2/OlaHsd, and B6.129P2-*Aim2*^Gt(*CSG445*)*Byg*^/J mice was also analyzed by MUGA (Neogen) (https://www.med.unc.edu/mmrrc/genotypes). Data from the full MUGA panel analysis of the AIM2 knockout mice are available upon request.

### Cell fractionation and intracellular localization.

NR9456 cells stably expressing myc-tagged Ifi205 (Ifi205myc) and NIH 3T3 cells stably expressing either Ifi205myc or HA-tagged Aim2 (Aim2HA) were established by transduction with retroviral vectors. Cells were fractionated by the modified rapid, efficient, and practical (REAP) method as previously described (67). The purity of fractions was determined by Western blotting with antibodies to β-tubulin (cytoplasmic fraction) and lamin B1 (nuclear fraction).

### Measurement of retrotransposon DNA.

Total and cytosolic DNA isolated from the cytoplasmic fraction by the REAP method was purified with DNeasy kits (Qiagen). Retrotransposon copy numbers were measured by qPCR and normalized to the mitochondrial gene for mtCytb. Mitochondrial DNA levels did not change upon knockdown of the different genes (Fig. S4). The sequences of the primers used are provided in Table S1.

### Raltegravir treatment.

NR9456 cells were treated with raltegravir, a reverse transcriptase inhibitor, at 0, 0.5, or 5 μg/ml. Cells were transfected with the siRNAs indicated 2 days after the initiation of raltegravir treatment. Raltegravir was kept in the medium throughout siRNA transfection.

### DNA pulldown assay.

NIH 3T3 cells were transfected with the expression plasmids indicated by using Lipofectamine 3000 (Invitrogen). The cells were fixed with 0.75% formaldehyde--phosphate-buffered saline (PBS) at 48 h posttransfection, and fixation was quenched with 125 mM glycine. Cells were washed with PBS and lysed with 0.1% NP-40--PBS. The cytoplasmic fraction was isolated by the REAP method and subjected to immunoprecipitation with the antibody-conjugated agarose beads indicated. Five percent of each lysate was aliquoted as the input. DNA was isolated from the beads by phenol-chloroform-isoamyl alcohol (25:24:1) extraction and isopropanol precipitation. Immunoprecipitated DNAs were measured by qPCR, and the values were normalized to the input DNA values.

### **Co**-**IP assay.**

HEK293T cells were transfected with the expression plasmids indicated and harvested with radioimmunoprecipitation assay buffer (50 mM Tris [pH 7.4], 150 mM NaCl, 1 mM EDTA, 1% Triton X-100, 1% deoxycholate, 0.1% SDS) at 48 h posttransfection. Lysates were immunoprecipitated with the antibody-conjugated agarose beads indicated at 4°C overnight. Immunoprecipitated proteins were eluted with 2× Laemmli sample loading buffer and analyzed by Western blotting. Ten percent of each lysates was analyzed as the input.

### Western blotting.

Samples were subjected to 10% sodium dodecyl sulfate-polyacrylamide gel electrophoresis and then transferred onto polyvinylidene difluoride membrane. The membrane was blocked with 5% skim milk or bovine serum albumin and reacted with primary antibodies (anti-myc tag [9B11 and 71D10], anti-HA [6E2 and C29F4], anti-AIM2 [catalog number 13095], anti-STING [D2P2F], anti-lamin B1 [D4Q4Z], anti-GAPDH [14C10; Cell Signaling Technology, Inc.], anti-α-tubulin, anti-TREX1 [SAB1410179; Sigma], and anti-V5 tag [Thermo Fisher Scientific] antibodies) and horseradish peroxidase-conjugated secondary antibodies (Cell Signaling Technology, Inc.). ECL Western blotting detection reagents (GE Healthcare Life Science) were used to detect the signals.

### PLA.

NR9456 cells cultured in eight-well chamber slides (Millipore) were fixed with 4% paraformaldehyde–PBS and permeabilized with 0.5% Triton X-100–PBS. Blocking and staining were performed with Duolink *in situ* PLA probes and detection reagents (Sigma). Fluorescence was analyzed with a BZ-X710-All-in-One fluorescence microscope (Keyence). Pictures were taken by using the Z-stacking function of the microscope and combined with a BZ-X analyzer. PLA dots were counted by ImageJ, and the values were normalized to the number of cells in the pictures. Thirty-three to 190 cells from 4 to 11 different pictures were analyzed for each condition. The primary antibodies used were anti-myc tag (Active Motif, Inc., 4E12), anti-AIM2 (Cell Signaling Technology, Inc., catalog number 13095), and anti-STING (Thermo Fischer Scientific PA5-23381 or Santa Cruz sc-241049) antibodies.

### cGAMP treatment.

NR9456 cells were subjected to siRNA knockdown as described above. Cells were transfected with 250 ng/ml or 4 μg/ml cGAMP (InvivoGen) by using Lipofectamine 2000 (Invitrogen) 4 h before harvest.

### Statistics.

Each experiment was done with three technical replicates per experiment, except where indicated otherwise in the figure legends. The data shown are the average of at least three independent experiments, as indicated in the figure legends. Statistical analysis was performed with GraphPad Prism software. Student’s *t* test was used for all comparisons.

### Accession number(s).

The data obtained in this study are publicly available in the NCBI database under accession number KY113153.

## References

[1] Liu J, Qian C, Cao X 2016 Post-translational modification control of innate immunity. Immunity 45:15–30. doi:10.1016/j.immuni.2016.06.020. 27438764

[2] Luecke S, Paludan SR 14 10 2016 Molecular requirements for sensing of intracellular microbial nucleic acids by the innate immune system. Cytokine. doi:10.1016/j.cyto.2016.10.003. 27751656

[3] Stetson DB, Ko JS, Heidmann T, Medzhitov R 2008 Trex1 prevents cell-intrinsic initiation of autoimmunity. Cell 134:587–598. doi:10.1016/j.cell.2008.06.032. 18724932 PMC2626626

[4] Brégnard C, Guerra J, Dejardin S, Passalacqua F, Benkirane M, Laguette N 2016 Upregulated LINE-1 activity in the Fanconi anemia cancer susceptibility syndrome leads to spontaneous pro-inflammatory cytokine production. EBioMedicine 8:184–194. 27428429 10.1016/j.ebiom.2016.05.005 PMC4919473

[5] Gifford R, Tristem M 2003 The evolution, distribution and diversity of endogenous retroviruses. Virus Genes 26:291–315. doi:10.1023/A:1024455415443. 12876457

[6] Stoye JP 2012 Studies of endogenous retroviruses reveal a continuing evolutionary saga. Nat Rev Microbiol 10:395–406. doi:10.1038/nrmicro2783. 22565131

[7] Pizarro JG, Cristofari G 2016 Post-transcriptional control of LINE-1 retrotransposition by cellular host factors in somatic cells. Front Cell Dev Biol 4:14. 27014690 10.3389/fcell.2016.00014 PMC4782638

[8] Mazur DJ, Perrino FW 1999 Identification and expression of the TREX1 and TREX2 cDNA sequences encoding mammalian 3′→5′ exonucleases. J Biol Chem 274:19655–19660. doi:10.1074/jbc.274.28.19655. 10391904

[9] Morita M, Stamp G, Robins P, Dulic A, Rosewell I, Hrivnak G, Daly G, Lindahl T, Barnes DE 2004 Gene-targeted mice lacking the Trex1 (DNase III) 3′→5′ DNA exonuclease develop inflammatory myocarditis. Mol Cell Biol 24:6719–6727. doi:10.1128/MCB.24.15.6719-6727.2004. 15254239 PMC444847

[10] Peschke K, Achleitner M, Frenzel K, Gerbaulet A, Ada SR, Zeller N, Lienenklaus S, Lesche M, Poulet C, Naumann R, Dahl A, Ravens U, Günther C, Müller W, Knobeloch KP, Prinz M, Roers A, Behrendt R 2016 Loss of Trex1 in dendritic cells is sufficient to trigger systemic autoimmunity. J Immunol 197:2157–2166. doi:10.4049/jimmunol.1600722. 27511730

[11] Lee-Kirsch MA, Gong M, Chowdhury D, Senenko L, Engel K, Lee YA, de Silva U, Bailey SL, Witte T, Vyse TJ, Kere J, Pfeiffer C, Harvey S, Wong A, Koskenmies S, Hummel O, Rohde K, Schmidt RE, Dominiczak AF, Gahr M, Hollis T, Perrino FW, Lieberman J, Hübner N 2007 Mutations in the gene encoding the 3′-5′ DNA exonuclease TREX1 are associated with systemic lupus erythematosus. Nat Genet 39:1065–1067. doi:10.1038/ng2091. 17660818

[12] Crow YJ, Hayward BE, Parmar R, Robins P, Leitch A, Ali M, Black DN, van Bokhoven H, Brunner HG, Hamel BC, Corry PC, Cowan FM, Frints SG, Klepper J, Livingston JH, Lynch SA, Massey RF, Meritet JF, Michaud JL, Ponsot G, Voit T, Lebon P, Bonthron DT, Jackson AP, Barnes DE, Lindahl T 2006 Mutations in the gene encoding the 3′-5′ DNA exonuclease TREX1 cause Aicardi-Goutières syndrome at the AGS1 locus. Nat Genet 38:917–920. doi:10.1038/ng1845. 16845398

[13] Crow YJ, Manel N 2015 Aicardi-Goutières syndrome and the type I interferonopathies. Nat Rev Immunol 15:429–440. doi:10.1038/nri3850. 26052098

[14] Ablasser A, Goldeck M, Cavlar T, Deimling T, Witte G, Röhl I, Hopfner KP, Ludwig J, Hornung V 2013 cGAS produces a 2′-5′-linked cyclic dinucleotide second messenger that activates STING. Nature 498:380–384. doi:10.1038/nature12306. 23722158 PMC4143541

[15] Wu J, Sun L, Chen X, Du F, Shi H, Chen C, Chen ZJ 2013 Cyclic GMP-AMP is an endogenous second messenger in innate immune signaling by cytosolic DNA. Science 339:826–830. doi:10.1126/science.1229963. 23258412 PMC3855410

[16] Sun L, Wu J, Du F, Chen X, Chen ZJ 2013 Cyclic GMP-AMP synthase is a cytosolic DNA sensor that activates the type I interferon pathway. Science 339:786–791. doi:10.1126/science.1232458. 23258413 PMC3863629

[17] Gao D, Wu J, Wu YT, Du F, Aroh C, Yan N, Sun L, Chen ZJ 2013 Cyclic GMP-AMP synthase is an innate immune sensor of HIV and other retroviruses. Science 341:903–906. doi:10.1126/science.1240933. 23929945 PMC3860819

[18] Ishikawa H, Ma Z, Barber GN 2009 STING regulates intracellular DNA-mediated, type I interferon-dependent innate immunity. Nature 461:788–792. doi:10.1038/nature08476. 19776740 PMC4664154

[19] Reference deleted.

[20] Reference deleted.

[21] Gao D, Li T, Li XD, Chen X, Li QZ, Wight-Carter M, Chen ZJ 2015 Activation of cyclic GMP-AMP synthase by self-DNA causes autoimmune diseases. Proc Natl Acad Sci U S A 112:E5699–E5705. doi:10.1073/pnas.1516465112. PMC4620884 26371324

[22] Gray EE, Treuting PM, Woodward JJ, Stetson DB 2015 Cutting edge: cGAS is required for lethal autoimmune disease in the Trex1-deficient mouse model of Aicardi-Goutières syndrome. J Immunol 195:1939–1943. doi:10.4049/jimmunol.1500969. 26223655 PMC4546858

[23] Albrecht M, Choubey D, Lengauer T 2005 The HIN domain of IFI-200 proteins consists of two OB folds. Biochem Biophys Res Commun 327:679–687. doi:10.1016/j.bbrc.2004.12.056. 15649401

[24] Asefa B, Klarmann KD, Copeland NG, Gilbert DJ, Jenkins NA, Keller JR 2004 The interferon-inducible p200 family of proteins: a perspective on their roles in cell cycle regulation and differentiation. Blood Cells Mol Dis 32:155–167. doi:10.1016/j.bcmd.2003.10.002. 14757431

[25] Jin T, Perry A, Jiang J, Smith P, Curry JA, Unterholzner L, Jiang Z, Horvath G, Rathinam VA, Johnstone RW, Hornung V, Latz E, Bowie AG, Fitzgerald KA, Xiao TS 2012 Structures of the HIN domain:DNA complexes reveal ligand binding and activation mechanisms of the AIM2 inflammasome and IFI16 receptor. Immunity 36:561–571. doi:10.1016/j.immuni.2012.02.014. 22483801 PMC3334467

[26] Brunette RL, Young JM, Whitley DG, Brodsky IE, Malik HS, Stetson DB 2012 Extensive evolutionary and functional diversity among mammalian AIM2-like receptors. J Exp Med 209:1969–1983. doi:10.1084/jem.20121960. 23045604 PMC3478938

[27] Gray EE, Winship D, Snyder JM, Child SJ, Geballe AP, Stetson DB 2016 The AIM2-like receptors are dispensable for the interferon response to intracellular DNA. Immunity 45:255–266. doi:10.1016/j.immuni.2016.06.015. 27496731 PMC4988931

[28] Li H, Wang ZX, Wu JW 2013 Comparative purification and characterization of two HIN domains, hematopoietic interferon-inducible nuclear antigens with a 200-amino-acid repeat, in murine AIM2-like receptors. Biosci Biotechnol Biochem 77:2283–2287. doi:10.1271/bbb.130544. 24200794

[29] Cridland JA, Curley EZ, Wykes MN, Schroder K, Sweet MJ, Roberts TL, Ragan MA, Kassahn KS, Stacey KJ 2012 The mammalian PYHIN gene family: phylogeny, evolution and expression. BMC Evol Biol 12:140. doi:10.1186/1471-2148-12-140. 22871040 PMC3458909

[30] Khare S, Ratsimandresy RA, de Almeida L, Cuda CM, Rellick SL, Misharin AV, Wallin MC, Gangopadhyay A, Forte E, Gottwein E, Perlman H, Reed JC, Greaves DR, Dorfleutner A, Stehlik C 2014 The pyrin domain-only protein POP3 inhibits ALR inflammasomes and regulates responses to infection with DNA viruses. Nat Immunol 15:343–353. doi:10.1038/ni.2829. 24531343 PMC4123781

[31] Hornung V, Ablasser A, Charrel-Dennis M, Bauernfeind F, Horvath G, Caffrey DR, Latz E, Fitzgerald KA 2009 AIM2 recognizes cytosolic dsDNA and forms a caspase-1-activating inflammasome with ASC. Nature 458:514–518. doi:10.1038/nature07725. 19158675 PMC2726264

[32] Fernandes-Alnemri T, Yu JW, Datta P, Wu J, Alnemri ES 2009 AIM2 activates the inflammasome and cell death in response to cytoplasmic DNA. Nature 458:509–513. doi:10.1038/nature07710. 19158676 PMC2862225

[33] Orzalli MH, Broekema NM, Diner BA, Hancks DC, Elde NC, Cristea IM, Knipe DM 2015 cGAS-mediated stabilization of IFI16 promotes innate signaling during herpes simplex virus infection. Proc Natl Acad Sci U S A 112:E1773–E1781. doi:10.1073/pnas.1424637112. 25831530 PMC4394261

[34] Kerur N, Veettil MV, Sharma-Walia N, Bottero V, Sadagopan S, Otageri P, Chandran B 2011 IFI16 acts as a nuclear pathogen sensor to induce the inflammasome in response to Kaposi sarcoma-associated herpesvirus infection. Cell Host Microbe 9:363–375. doi:10.1016/j.chom.2011.04.008. 21575908 PMC3113467

[35] Monroe KM, Yang Z, Johnson JR, Geng X, Doitsh G, Krogan NJ, Greene WC 2014 IFI16 DNA sensor is required for death of lymphoid CD4 T cells abortively infected with HIV. Science 343:428–432. doi:10.1126/science.1243640. 24356113 PMC3976200

[36] Johnson KE, Bottero V, Flaherty S, Dutta S, Singh VV, Chandran B 2014 IFI16 restricts HSV-1 replication by accumulating on the HSV-1 genome, repressing HSV-1 gene expression, and directly or indirectly modulating histone modifications. PLoS Pathog 10:e1004503. doi:10.1371/journal.ppat.1004503. 25375629 PMC4223080

[37] Jakobsen MR, Bak RO, Andersen A, Berg RK, Jensen SB, Tengchuan J, Laustsen A, Hansen K, Ostergaard L, Fitzgerald KA, Xiao TS, Mikkelsen JG, Mogensen TH, Paludan SR 2013 IFI16 senses DNA forms of the lentiviral replication cycle and controls HIV-1 replication. Proc Natl Acad Sci U S A 110:E4571–E4580. doi:10.1073/pnas.1311669110. 24154727 PMC3845190

[38] Unterholzner L, Keating SE, Baran M, Horan KA, Jensen SB, Sharma S, Sirois CM, Jin T, Latz E, Xiao TS, Fitzgerald KA, Paludan SR, Bowie AG 2010 IFI16 is an innate immune sensor for intracellular DNA. Nat Immunol 11:997–1004. doi:10.1038/ni.1932. 20890285 PMC3142795

[39] Stavrou S, Blouch K, Kotla S, Bass A, Ross SR 2015 Nucleic acid recognition orchestrates the anti-viral response to retroviruses. Cell Host Microbe 17:478–488. doi:10.1016/j.chom.2015.02.021. 25816774 PMC4393365

[40] Stavrou S, Nitta T, Kotla S, Ha D, Nagashima K, Rein AR, Fan H, Ross SR 2013 Murine leukemia virus glycosylated Gag blocks apolipoprotein B editing complex 3 and cytosolic sensor access to the reverse transcription complex. Proc Natl Acad Sci U S A 110:9078–9083. doi:10.1073/pnas.1217399110. 23671100 PMC3670389

[41] Cuevas CD, Lavanya M, Wang E, Ross SR 2011 Junin virus infects mouse cells and induces innate immune responses. J Virol 85:11058–11068. doi:10.1128/JVI.05304-11. 21880772 PMC3194972

[42] Beck-Engeser GB, Eilat D, Harrer T, Jäck HM, Wabl M 2009 Early onset of autoimmune disease by the retroviral integrase inhibitor raltegravir. Proc Natl Acad Sci U S A 106:20865–20870. doi:10.1073/pnas.0908074106. 19923437 PMC2791572

[43] Koh Y, Matreyek KA, Engelman A 2011 Differential sensitivities of retroviruses to integrase strand transfer inhibitors. J Virol 85:3677–3682. doi:10.1128/JVI.02541-10. 21270168 PMC3067883

[44] Tannenbaum CS, Major J, Ohmori Y, Hamilton TA 1993 A lipopolysaccharide-inducible macrophage gene (D3) is a new member of an interferon-inducible gene cluster and is selectively expressed in mononuclear phagocytes. J Leukoc Biol 53:563–568. 7684766 10.1002/jlb.53.5.563

[45] Zhang K, Kagan D, DuBois W, Robinson R, Bliskovsky V, Vass WC, Zhang S, Mock BA 2009 Mndal, a new interferon-inducible family member, is highly polymorphic, suppresses cell growth, and may modify plasmacytoma susceptibility. Blood 114:2952–2960. doi:10.1182/blood-2009-01-198812. 19654412 PMC2756205

[46] Reference deleted.

[47] Rathinam VAK, Jiang Z, Waggoner SN, Sharma S, Cole LE, Waggoner L, Vanaja SK, Monks BG, Ganesan S, Latz E, Hornung V, Vogel SN, Szomolanyi-Tsuda E, Fitzgerald KA 2010 The AIM2 inflammasome is essential for host defense against cytosolic bacteria and DNA viruses. Nat Immunol 11:395–402. doi:10.1038/ni.1864. 20351692 PMC2887480

[48] Rice GI, Bond J, Asipu A, Brunette RL, Manfield IW, Carr IM, Fuller JC, Jackson RM, Lamb T, Briggs TA, Ali M, Gornall H, Couthard LR, Aeby A, Attard-Montalto SP, Bertini E, Bodemer C, Brockmann K, Brueton LA, Corry PC, Desguerre I, Fazzi E, Cazorla AG, Gener B, Hamel BC, Heiberg A, Hunter M, van der Knaap MS, Kumar R, Lagae L, Landrieu PG, Lourenco CM, Marom D, McDermott MF, van der Merwe W, Orcesi S, Prendiville JS, Rasmussen M, Shalev SA, Soler DM, Shinawi M, Spiegel R, Tan TY, Vanderver A, Wakeling EL, Wassmer E, Whittaker E, Lebon P, Stetson DB, Bonthron DT, Crow YJ 2009 Mutations involved in Aicardi-Goutières syndrome implicate SAMHD1 as regulator of the innate immune response. Nat Genet 41:829–832. doi:10.1038/ng.373. 19525956 PMC4154505

[49] Rice GI, del Toro Duany Y, Jenkinson EM, Forte GM, Anderson BH, Ariaudo G, Bader-Meunier B, Baildam EM, Battini R, Beresford MW, Casarano M, Chouchane M, Cimaz R, Collins AE, Cordeiro NJ, Dale RC, Davidson JE, De Waele L, Desguerre I, Faivre L, Fazzi E, Isidor B, Lagae L, Latchman AR, Lebon P, Li C, Livingston JH, Lourenço CM, Mancardi MM, Masurel-Paulet A, McInnes IB, Menezes MP, Mignot C, O’Sullivan J, Orcesi S, Picco PP, Riva E, Robinson RA, Rodriguez D, Salvatici E, Scott C, Szybowska M, Tolmie JL, Vanderver A, Vanhulle C, Vieira JP, Webb K, Whitney RN, Williams SG, Wolfe LA, Zuberi SM, Hur S, Crow YJ 2014 Gain-of-function mutations in IFIH1 cause a spectrum of human disease phenotypes associated with upregulated type I interferon signaling. Nat Genet 46:503–509. doi:10.1038/ng.2933. 24686847 PMC4004585

[50] Rice GI, Kasher PR, Forte GM, Mannion NM, Greenwood SM, Szynkiewicz M, Dickerson JE, Bhaskar SS, Zampini M, Briggs TA, Jenkinson EM, Bacino CA, Battini R, Bertini E, Brogan PA, Brueton LA, Carpanelli M, De Laet C, de Lonlay P, del Toro M, Desguerre I, Fazzi E, Garcia-Cazorla A, Heiberg A, Kawaguchi M, Kumar R, Lin JP, Lourenco CM, Male AM, Marques W Jr., Mignot C, Olivieri I, Orcesi S, Prabhakar P, Rasmussen M, Robinson RA, Rozenberg F, Schmidt JL, Steindl K, Tan TY, van der Merwe WG, Vanderver A, Vassallo G, Wakeling EL, Wassmer E, Whittaker E, Livingston JH, Lebon P, Suzuki T, McLaughlin PJ, Keegan LP, O’Connell MA, Lovell SC, Crow YJ 2012 Mutations in ADAR1 cause Aicardi-Goutières syndrome associated with a type I interferon signature. Nat Genet 44:1243–1248. doi:10.1038/ng.2414. 23001123 PMC4154508

[51] Crow YJ, Leitch A, Hayward BE, Garner A, Parmar R, Griffith E, Ali M, Semple C, Aicardi J, Babul-Hirji R, Baumann C, Baxter P, Bertini E, Chandler KE, Chitayat D, Cau D, Déry C, Fazzi E, Goizet C, King MD, Klepper J, Lacombe D, Lanzi G, Lyall H, Martínez-Frías ML, Mathieu M, McKeown C, Monier A, Oade Y, Quarrell OW, Rittey CD, Rogers RC, Sanchis A, Stephenson JB, Tacke U, Till M, Tolmie JL, Tomlin P, Voit T, Weschke B, Woods CG, Lebon P, Bonthron DT, Ponting CP, Jackson AP 2006 Mutations in genes encoding ribonuclease H2 subunits cause Aicardi-Goutières syndrome and mimic congenital viral brain infection. Nat Genet 38:910–916. doi:10.1038/ng1842. 16845400

[52] Beck-Engeser GB, Eilat D, Wabl M 2011 An autoimmune disease prevented by anti-retroviral drugs. Retrovirology 8:91. doi:10.1186/1742-4690-8-91. 22067273 PMC3264515

[53] Bürckstümmer T, Baumann C, Blüml S, Dixit E, Dürnberger G, Jahn H, Planyavsky M, Bilban M, Colinge J, Bennett KL, Superti-Furga G 2009 An orthogonal proteomic-genomic screen identifies AIM2 as a cytoplasmic DNA sensor for the inflammasome. Nat Immunol 10:266–272. doi:10.1038/ni.1702. 19158679

[54] Liu F, Jiao Y, Zhu Z, Sun C, Li H 2014 Interferon-inducible protein 205 (p205) plays a role in adipogenic differentiation of mouse adipose-derived stem cells. Mol Cell Endocrinol 392:80–89. doi:10.1016/j.mce.2014.05.009. 24859602

[55] Asefa B, Dermott JM, Kaldis P, Stefanisko K, Garfinkel DJ, Keller JR 2006 p205, a potential tumor suppressor, inhibits cell proliferation via multiple pathways of cell cycle regulation. FEBS Lett 580:1205–1214. doi:10.1016/j.febslet.2006.01.032. 16458891

[56] Corrales L, Woo SR, Williams JB, McWhirter SM, Dubensky TW Jr., Gajewski TF 2016 Antagonism of the STING pathway via activation of the AIM2 inflammasome by intracellular DNA. J Immunol 196:3191–3198. doi:10.4049/jimmunol.1502538. 26927800 PMC4800192

[57] Liu C, Yue R, Yang Y, Cui Y, Yang L, Zhao D, Zhou X 2016 AIM2 inhibits autophagy and IFN-beta production during M. bovis infection. Oncotarget 7:46972–46987. 27409673 10.18632/oncotarget.10503 PMC5216917

[58] Storek KM, Gertsvolf NA, Ohlson MB, Monack DM 2015 cGAS and Ifi204 cooperate to produce type I IFNs in response to Francisella infection. J Immunol 194:3236–3245. doi:10.4049/jimmunol.1402764. 25710914 PMC4367159

[59] Choubey D, Panchanathan R 2008 Interferon-inducible Ifi200-family genes in systemic lupus erythematosus. Immunol Lett 119:32–41. doi:10.1016/j.imlet.2008.06.001. 18598717 PMC2585765

[60] Weischenfeldt J, Porse B 2008 Bone marrow-derived macrophages (BMM): isolation and applications. CSH Protoc 2008:pdb.prot5080. doi:10.1101/pdb.prot5080. 21356739

[61] Dobin A, Davis CA, Schlesinger F, Drenkow J, Zaleski C, Jha S, Batut P, Chaisson M, Gingeras TR 2013 STAR: ultrafast universal RNA-seq aligner. Bioinformatics 29:15–21. doi:10.1093/bioinformatics/bts635. 23104886 PMC3530905

[62] Keane TM, Goodstadt L, Danecek P, White MA, Wong K, Yalcin B, Heger A, Agam A, Slater G, Goodson M, Furlotte NA, Eskin E, Nellåker C, Whitley H, Cleak J, Janowitz D, Hernandez-Pliego P, Edwards A, Belgard TG, Oliver PL, McIntyre RE, Bhomra A, Nicod J, Gan X, Yuan W, van der Weyden L, Steward CA, Bala S, Stalker J, Mott R, Durbin R, Jackson IJ, Czechanski A, Guerra-Assunção JA, Donahue LR, Reinholdt LG, Payseur BA, Ponting CP, Birney E, Flint J, Adams DJ 2011 Mouse genomic variation and its effect on phenotypes and gene regulation. Nature 477:289–294. doi:10.1038/nature10413. 21921910 PMC3276836

[63] Church DM, Schneider VA, Graves T, Auger K, Cunningham F, Bouk N, Chen HC, Agarwala R, McLaren WM, Ritchie GR, Albracht D, Kremitzki M, Rock S, Kotkiewicz H, Kremitzki C, Wollam A, Trani L, Fulton L, Fulton R, Matthews L, Whitehead S, Chow W, Torrance J, Dunn M, Harden G, Threadgold G, Wood J, Collins J, Heath P, Griffiths G, Pelan S, Grafham D, Eichler EE, Weinstock G, Mardis ER, Wilson RK, Howe K, Flicek P, Hubbard T 2011 Modernizing reference genome assemblies. PLoS Biol 83:e1001091. doi:10.1371/journal.pbio.1001091. PMC3130012 21750661

[64] Stanke M, Schöffmann O, Morgenstern B, Waack S 2006 Gene prediction in eukaryotes with a generalized hidden Markov model that uses hints from external sources. BMC Bioinformatics 7:62. doi:10.1186/1471-2105-7-62. 16469098 PMC1409804

[65] Dietrich WF, Miller J, Steen R, Merchant MA, Damron-Boles D, Husain Z, Dredge R, Daly MJ, Ingalls KA, O’Connor TJ 1996 A comprehensive genetic map of the mouse genome. Nature 380:149–152. doi:10.1038/380149a0. 8600386

[66] Okeoma CM, Petersen J, Ross SR 2009 Expression of murine APOBEC3 alleles in different mouse strains and their effect on mouse mammary tumor virus infection. J Virol 83:3029–3038. doi:10.1128/JVI.02536-08. 19153233 PMC2655539

[67] Suzuki K, Bose P, Leong-Quong RY, Fujita DJ, Riabowol K 2010 REAP: a two minute cell fractionation method. BMC Res Notes 3:294. doi:10.1186/1756-0500-3-294. 21067583 PMC2993727

[68] Huang Y, Zhang L 2004 Rapid and sensitive dot-matrix methods for genome analysis. Bioinformatics 20:460–466. doi:10.1093/bioinformatics/btg429. 14764561

