# Supplementary material for: AIM2-Like Receptors Positively and Negatively Regulate the Interferon Response Induced by Cytosolic DNA
Source: mBio. 2017 Jul 5;8(4):e00944-17. doi: 10.1128/mBio.00944-17 (PMC5573678; doi:10.1128/mBio.00944-17)
Supplement: FIG S1 [file mbo003173364sf1.pdf]

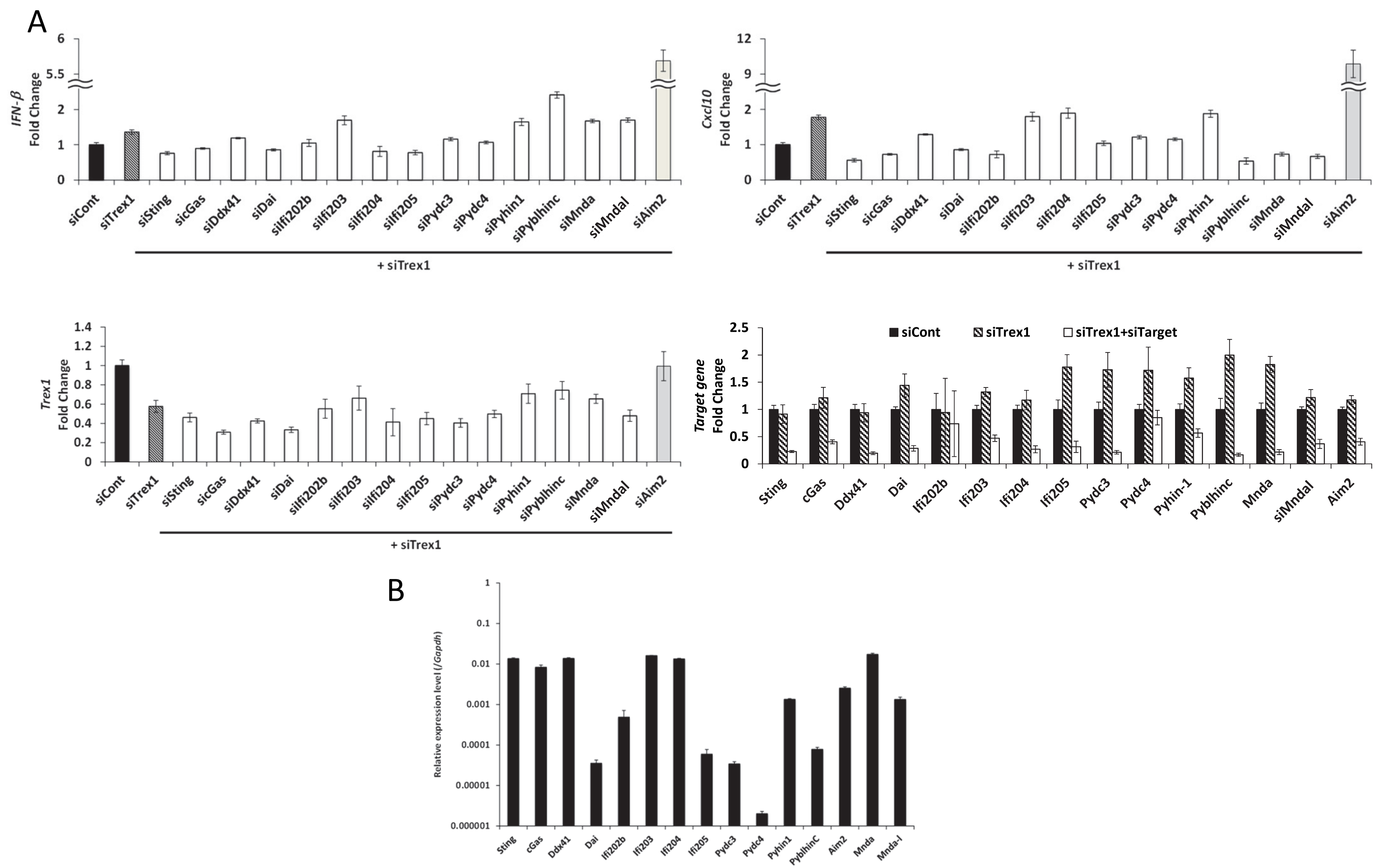

Fig. S1. Initial screening of the molecules involved in the type I IFN response upon Trex1 knockdown. A) NR9456 cells were transfected with siRNAs as indicated. Changes of expression levels of indicated genes were measured by RT-qPCR. Values were normalized to *Gapdh* and are shown as mean ± SEM of six experiments for siCont and siTrex1+siAim2 and three experiments for the others performed with duplicate technical replicates. B) Basal gene expression levels were measured by RT-qPCR in NR9456 cells. Values were normalized to *Gapdh* and are shown as mean ± SEM of three different cultures.
