# Supplementary material for: AIM2-Like Receptors Positively and Negatively Regulate the Interferon Response Induced by Cytosolic DNA
Source: mBio. 2017 Jul 5;8(4):e00944-17. doi: 10.1128/mBio.00944-17 (PMC5573678; doi:10.1128/mBio.00944-17)
Supplement: FIG S2 [file mbo003173364sf2.pdf]

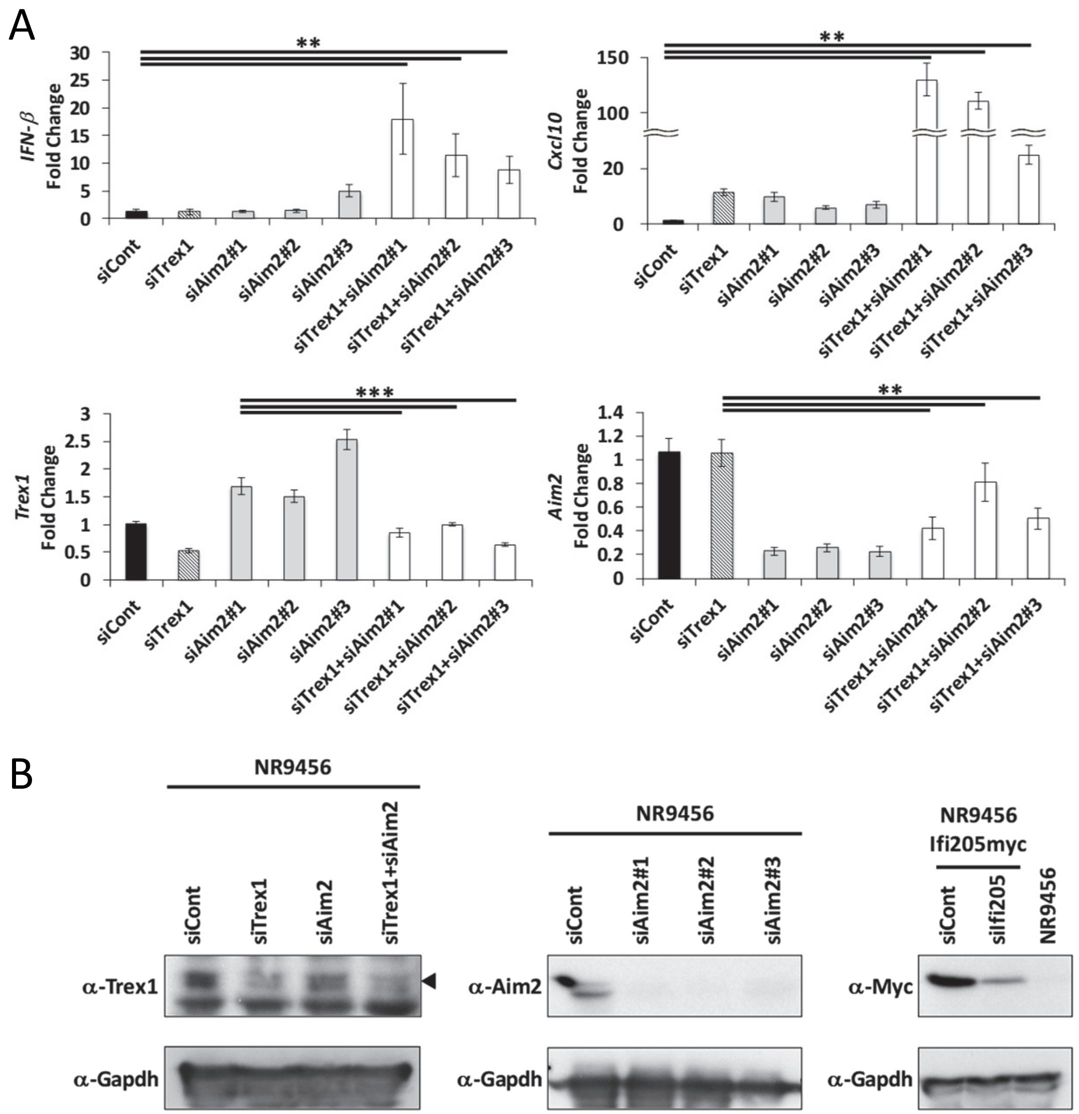

Fig. S2. The effect of siAim2 in IFN response was not off-target. A) NR9456 cells were transfected with the indicated siRNAs and expression levels of *IFN-b*, *Cxcl10*, *Trex1* and *Aim2* measured by RT-qPCR. Values were normalized to *Gapdh* and are shown as mean ± SEM of three experiments. *P<0.05, **P<0.005 and ***P<0.0005 (two-tailed t-test). B) Knockdown effects of each siRNA in macrophages. NR9456 or NR9456-IFI205myc cells were transfected with indicated siRNAs. TREX1, AIM2 and IFI205myc were detected by the indicated antibodies in western blotting. Western blotting for TREX1, IFI205myc and AIM2 were performed 1, 1 and 2 times, respectively.
