# Supplementary material for: AIM2-Like Receptors Positively and Negatively Regulate the Interferon Response Induced by Cytosolic DNA
Source: mBio. 2017 Jul 5;8(4):e00944-17. doi: 10.1128/mBio.00944-17 (PMC5573678; doi:10.1128/mBio.00944-17)
Supplement: FIG S3 [file mbo003173364sf3.pdf]

	Fold change (vs. siCont)		
Symbol	siTrex1	siAim2	siTrex1+siAim2
**Aim2**	1.1678	0.2245	0.3033
**Mavs**	0.539	0.3885	0.4272
**Nlrp3**	0.6742	0.6798	0.4245
**Tlr8**	0.1815	0.4407	0.4654
**Ccl5**	0.9378	3.4801	3.0799
**Cxcl10**	1.5201	3.4925	10.1537
**Ddx58**	1.1812	1.6149	2.0546
**Dhx58**	2.4088	3.1169	2.3176
**Ifnb1**	1.0476	3.5128	8.1813
**Irf7**	1.4186	3.8604	6.5774
**Isg15**	2.0082	9.6459	20.9148
**Mx1**	2.2979	3.9929	10.9294
**Oas2**	1.1077	1.2195	2.2427
**Stat1**	1.4313	2.3169	2.6309

Fig. S3. Expression profiles of individual genes in the PCR array. Downregulated or upregulated genes are shown with blue or red colors, respectively.
