# Supplementary material for: AIM2-Like Receptors Positively and Negatively Regulate the Interferon Response Induced by Cytosolic DNA
Source: mBio. 2017 Jul 5;8(4):e00944-17. doi: 10.1128/mBio.00944-17 (PMC5573678; doi:10.1128/mBio.00944-17)
Supplement: FIG S4 [file mbo003173364sf4.pdf]

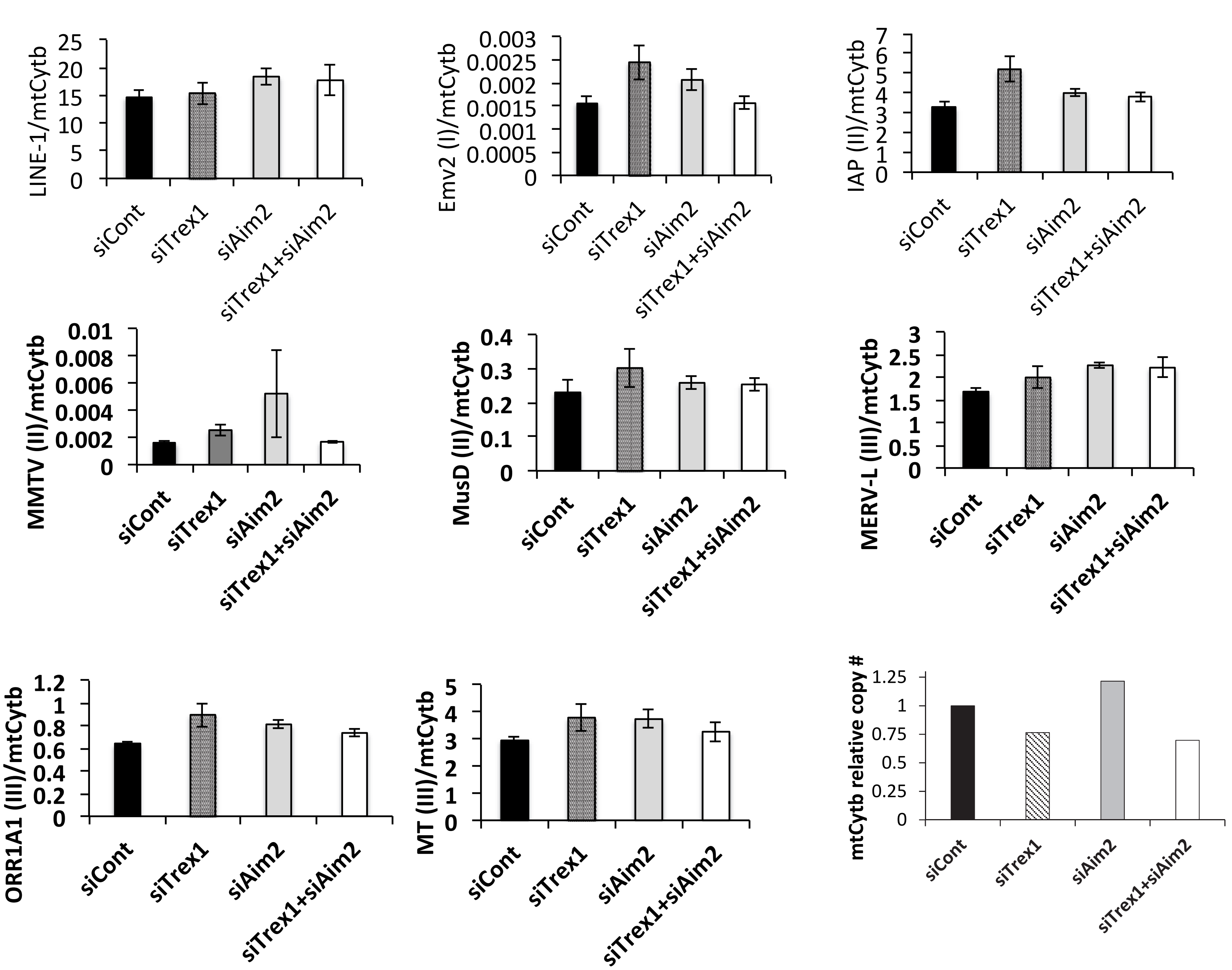

Fig. S4. Influence of *Trex1* and *Aim2* knockdown on endogenous retrotransposon copy numbers. NR9456 cells were transfected with siRNAs as indicated. Whole DNA was isolated 72 h after the first transfection to measure the copy numbers of endogenous retroviruses by qPCR and normalized to the mtCytb, whose copy numbers did not change upon Trex1 knockdown (final panel). Experiments were performed in triplicate and repeated 3 times.
