# Supplementary material for: AIM2-Like Receptors Positively and Negatively Regulate the Interferon Response Induced by Cytosolic DNA
Source: mBio. 2017 Jul 5;8(4):e00944-17. doi: 10.1128/mBio.00944-17 (PMC5573678; doi:10.1128/mBio.00944-17)
Supplement: FIG S5 [file mbo003173364sf5.pdf]

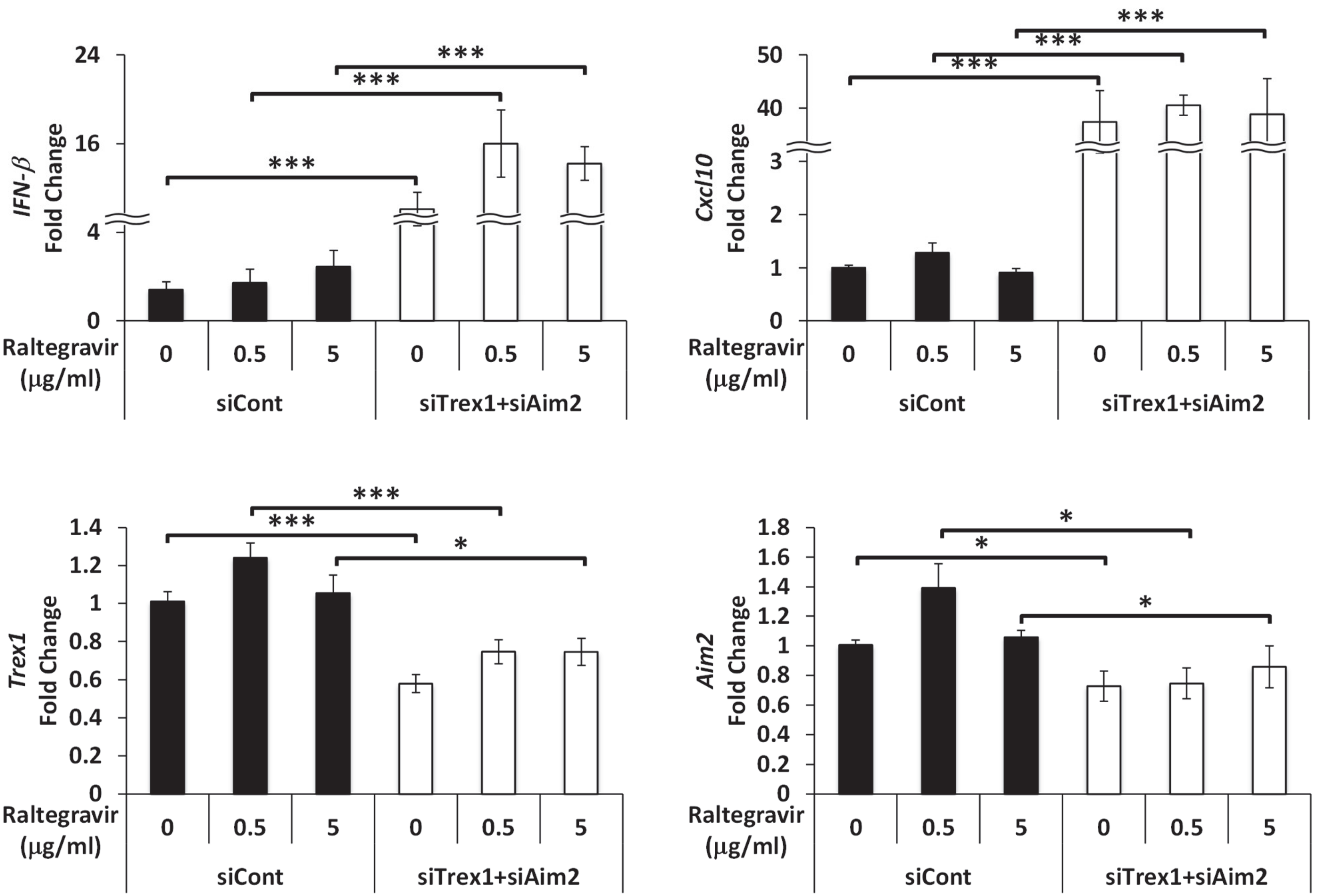

Fig. S5. Raltegravir does not increase the type I IFN response in macrophages. NR9456 cells treated with raltegravir at the different concentration (0, 0.5, 5 mg/ml) were sequentially transfected with indicated siRNAs. Expression levels of IFN-b, Cxcl10, Trex1 and Aim2 were measured by RT-qPCR. Values were normalized to *Gapdh* and are shown as mean ± SEM of three experiments in triplicate. *P<0.05 and ***P<0.0005 (two-tailed t-test).
