# Supplementary material for: AIM2-Like Receptors Positively and Negatively Regulate the Interferon Response Induced by Cytosolic DNA
Source: mBio. 2017 Jul 5;8(4):e00944-17. doi: 10.1128/mBio.00944-17 (PMC5573678; doi:10.1128/mBio.00944-17)
Supplement: FIG S7 [file mbo003173364sf7.pdf]

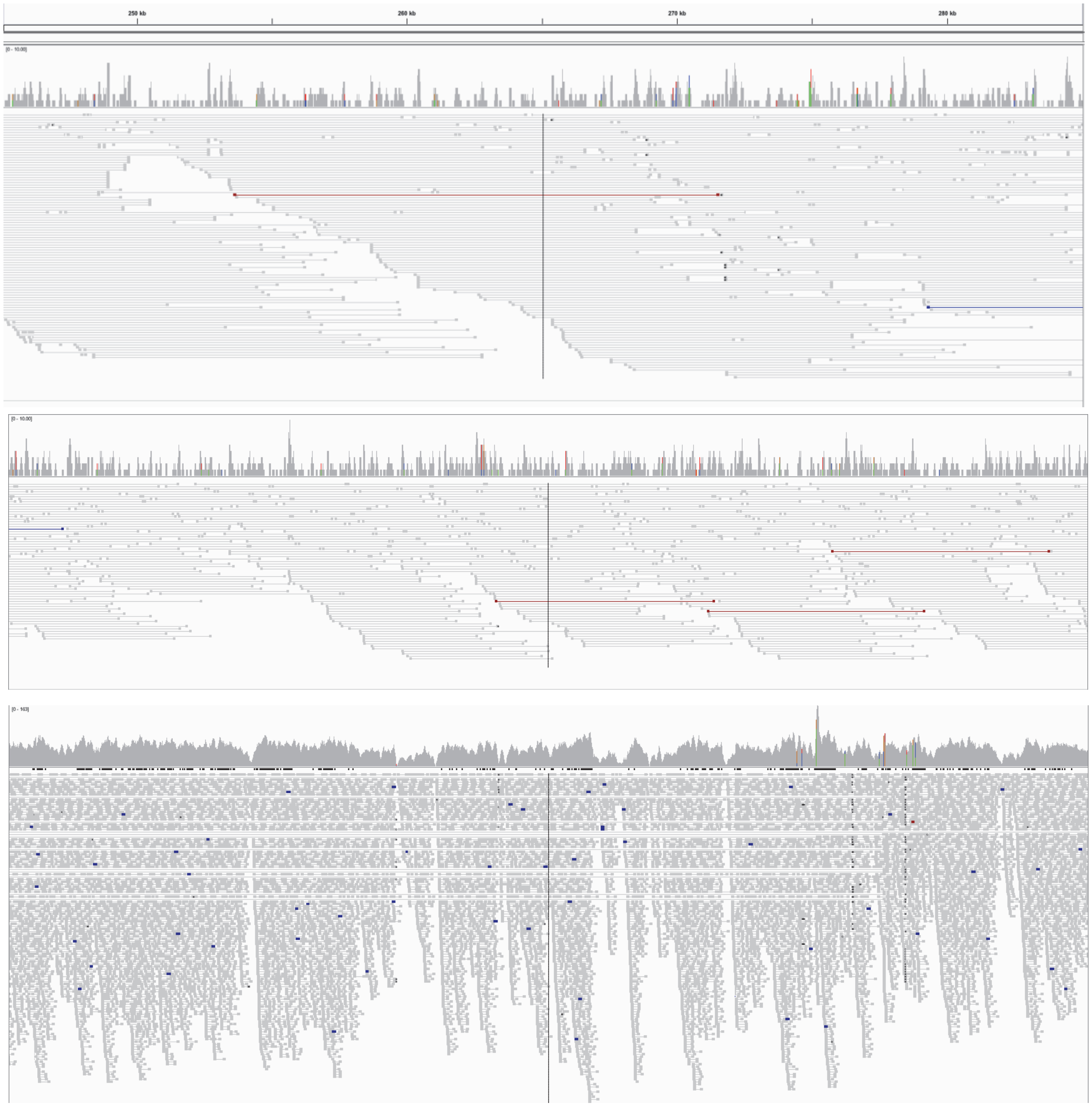

Fig. S7. Illumina reads mapped to the gap between two 129X1 BACs visualized by IGV. From top to bottom are 10Kb, 6Kb and short insertion Illumina. Sequence from 273Kb to 278Kb contains transposon elements, which causes mismatches and mapping errors.
