# Supplementary material for: AIM2-Like Receptors Positively and Negatively Regulate the Interferon Response Induced by Cytosolic DNA
Source: mBio. 2017 Jul 5;8(4):e00944-17. doi: 10.1128/mBio.00944-17 (PMC5573678; doi:10.1128/mBio.00944-17)
Supplement: FIG S8 [file mbo003173364sf8.pdf]

A

	Aim2	GM4955	Pyhin-B	Pydc4	Pyhin-1	Pydc3	PyhinA	IFI204	IFI203_1	IFI202_1	IFI203_2	IFI202_2	IFI203_3	Mndal	Mnda	IFI202_3	IFI205
BL6	1700	4000	500	5000	14000	200	10000	27000	7000	-	-	<100	-	18000	42000	-	40000
129	2400	1000	5000	600	10000	1100	16000	32000	12000	8000	8000	6000	4000	10000	-	6000	12000

B

	D1Mit113	D1Mit206	Aim2	pydc3	PyhinA	Ifi202*	D1Mit150	A3 (chr. 16)
Aim2 KO	129	129	-	129	129	129	129	BL/6

Fig. S8. Comparison of ALRs between C57BL/6 and 129 mice strains. (*A*) Profiles of IFNβ-mediated ALRs induction in BMDMs from different mice strains. Shown are the relative read counts from the RNAseq analysis (see Materials and Methods). (*B*) Origins of ALRs in Aim2 KO mice. *Both copies of IFI202 in 129 mice have a characteristic 400 bp deletion, resulting in a smaller PCR product.
