# Supplementary material for: AIM2-Like Receptors Positively and Negatively Regulate the Interferon Response Induced by Cytosolic DNA
Source: mBio. 2017 Jul 5;8(4):e00944-17. doi: 10.1128/mBio.00944-17 (PMC5573678; doi:10.1128/mBio.00944-17)
Supplement: FIG S9 [file mbo003173364sf9.pdf]

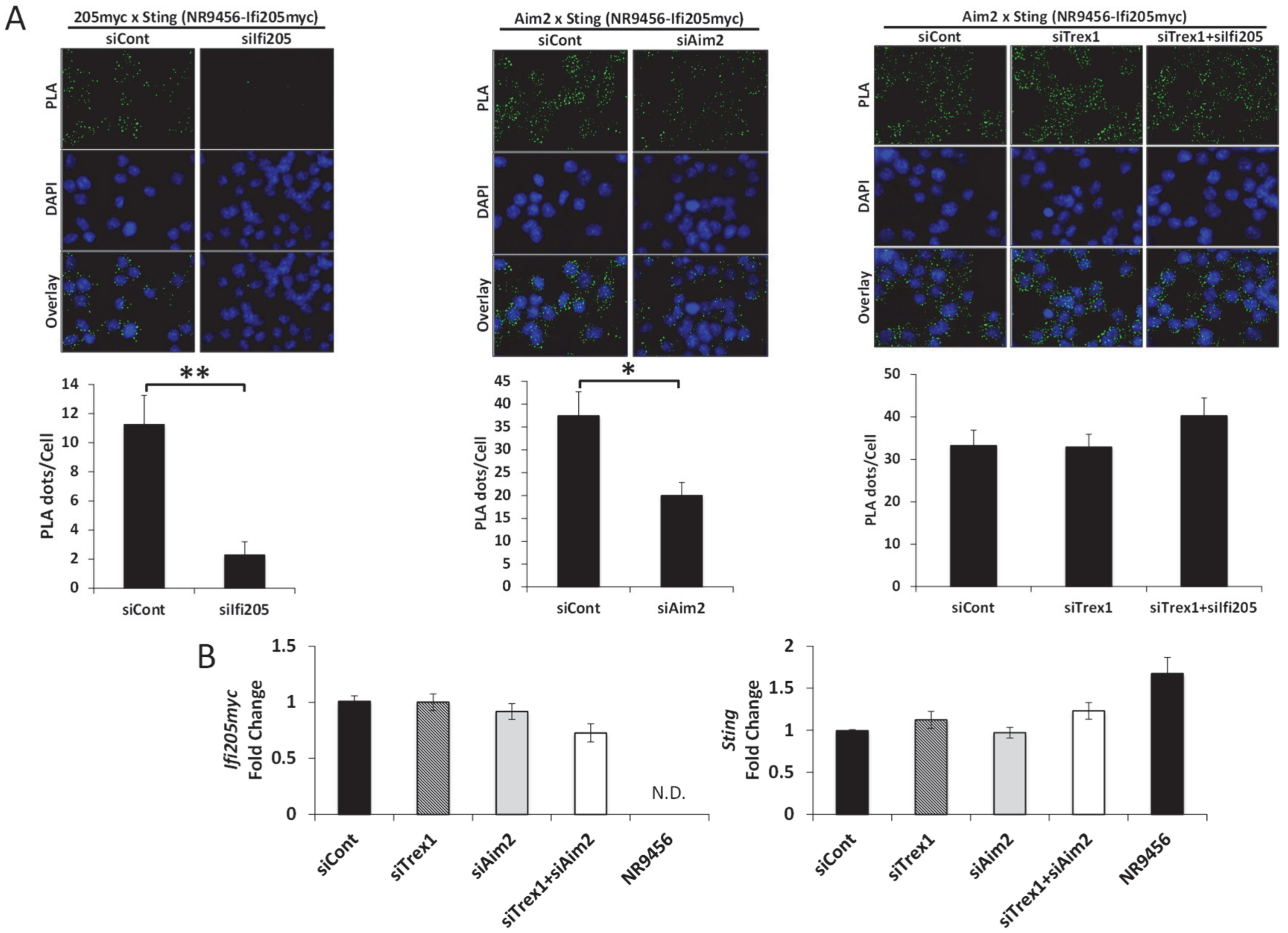

Fig S9. Knockdown effects of siRNAs in PLA. (*A*) NR9456-IFI205myc cells were transfected with indicated siRNAs to confirm the reduction of IFI205myc-STING or AIM2-STING interaction. PLA dots were counted and normalized to cell numbers based on DAPI staining. Values are shown as mean ± SEM of different pictures. Cells (pictures) numbers; 110 (5) and 156 (5) for siCont and siIfi205 of IFI205myc-STING, respectively; 92 (4) and 111 (4) for siCont and siAim2 of Aim2-STING, respectively; 103 (4), 96 (4) and 69 (4) for siCont, siTrex1 and siTrex1+siIfi205 of Aim2-STING, respectively. *P<0.05 and **P<0.005 (two-tailed t-test). (*B*) Expression levels of *Ifi205myc* and *Sting* measured by RT-qPCR upon knockdown of indicated genes. Values were normalized to *Gapdh* and are shown as mean ± SEM of 3 experiments.
