# Supplementary material for: AIM2-Like Receptors Positively and Negatively Regulate the Interferon Response Induced by Cytosolic DNA
Source: mBio. 2017 Jul 5;8(4):e00944-17. doi: 10.1128/mBio.00944-17 (PMC5573678; doi:10.1128/mBio.00944-17)
Supplement: TABLE S1 [file mbo003173364st1.docx]

**Table S1. Realtime PCR Primers (a) and siRNA sequences (b).**

**a) Primers**

**ALRs and sensors**

Trex1 CGTCAACGCTTCGATGACA/AGTCATAGCGGTCACCGTTGT

Sting AGGCGTCTGTATCCTGGAGT/CAAGTGTCCGGCAGAAGAGT

cGas TGAAACAGGTGCTTTCTATCTTGTG/GTGTTACAGCAGGGCTTCCT

Ddx41 ATTCCGGGAAGGCAAGAAGG/AGGCTTTGAGGTCCATGA

Dai GAGGACGCCACCATTGGTAA/GGAGTGGCTTCAGAGCTTGT

Ifi202b AGCTGACACACTCTGCCTTG/TGATTGAGTTCAAGCCGGGA

Ifi203 ATGTCAGGTGTGAACCAGGC/TACAGACCTCAGGAGGCACA

Ifi204 GGGGAGTGGAAAATGGCACA/GCACCATCACTTGTTTGGGA

Ifi205 A GCATCTGAAAGAGGCAAGAC/GAGGCTGATCTGCTTTCCCA

Ifi205Myc GGACAACCAAAGTTAGTGTG/CAGATCCTCTTCTGAGATG

Pydc3 GCCTGATGGAAGCTTGGGAA/CTGGGGAGTCAGTGGTTCAC

Pydc4 CCTCTGAACCCTGTCTGCCA/CCAGCATCCTCTGGGAACTTT

Pyhin1 TCTGGACCCTCCAGTGTCTT/ACCTTGCTGGTGACCATTTT

PyblhinC CGACAGCAATGTGATGAATATCC/TTCCTAGCCTGGATGACCTGC

Aim2 GTTGAATCTAACCACGAAGTCC/CTACAAGGTCCAGATTTCAACTG

Mnda TCTGAAAGAGGCGAGACTTCTG/TCTGGGAATGTTCTGGTTCTGG

Mnda‐l ACCCAGCAGTTCCTTAAACAAG/TTCAATCATTTGGTCAGGATCAG

**Cytokines and controls**

Ifnβ AAGAGTTACACTGCCTTTGCCACT/CACTGTCTGCTGGTGGAGTTCATC

Cxcl10 AAGTGCTGCCGTCATTTTCT/GTGGCAATGATCTCAACACG

Gapdh CCCCTTCATTGACCTCAACTACA/CGCTCCTGGAGGATGGTGAT

**Retrotransposons**

LINE‐1 TTTGGGACACAATGAAAGC/CTGCCGTCTACTCCTCTTG

Emv2 AGGCTGTTCCAGAGATTGTG/TTCTGGACCACCACACGAC

IAP AAGCAGCAATCACCCACTTTGG/CAATCATTAGATGCGGCTGCCAAG

MusD GATTGGTGGAAGTTTAGCTAGCAT/TAGCATTCTCATAAGCCAATTGCAT

MMTV C GTGAAAGACTCGCCAGAGCTA/CCTTATGTCAAAGGTATCCACAG

ORR1A1 CTTTAGTTGATGGCCCAGGA/CCAACTCTGCCCTCTGTAGC

MERV‐L CCCATCATGAGCTGGGTACT/CGTGCAGAGCCATCAGTAAA

MT ATGTCTTGGGGAGGACTGTG/AGCCCCAGCTAACCAGAACT

mtCytb CCATTCTACGCTCAATCCCCAAT/GGGCGGAATATTAGGCTTCGT

**Primers used for mapping the ALR locus**

Pydc3 GCCAAGACCTGATCAGACCAATCC/CTAAGAGGCTGCTCCCTATCTAGC

PyhinA GGATGGACTGCTGTGTACTGTTGC/GTAAAAGCATCCATGAAAGTCCAAGC

Ifi202 GGCATCATCTGAATTCCTTCCTC/GGAAAATCTTTCTTCTGCTTGGC

| **b) siRNAs used for gene knockdown** | | |
| --- | --- | --- |
| Gene | Cat# | Vendor |
| Trex1 | s75453 | Invitrogen |
| Aim2-1, -2, -3 | s234106, s234107, s234108 | Invitrogen |
| cGas | s103166 | Invitrogen |
| Sting | s91056 | Invitrogen |
| Ddx41 | s91394 | Invitrogen |
| Dai | s233870 | Invitrogen |
| Ifi202b | s233741 | Invitrogen |
| Ifi203 | s68051 | Invitrogen |
| Ifi204 | s68052 | Invitrogen |
| Ifi205 | s105482 | Invitrogen |
| Mnda | SR414083 | Origene |
| Mnda-l | SR416974 | Origene |
| Pydc3 | s127236 | Invitrogen |
| Pydc4 | s120496 | Invitrogen |
| Pyhin1 | s22643 | Invitrogen |
| PyblhinC | SR421002 | Origene |
