# Supplementary material for: AIM2-Like Receptors Positively and Negatively Regulate the Interferon Response Induced by Cytosolic DNA
Source: mBio. 2017 Jul 5;8(4):e00944-17. doi: 10.1128/mBio.00944-17 (PMC5573678; doi:10.1128/mBio.00944-17)
Supplement: FIG S6 [file mbo003173364sf6.pdf]

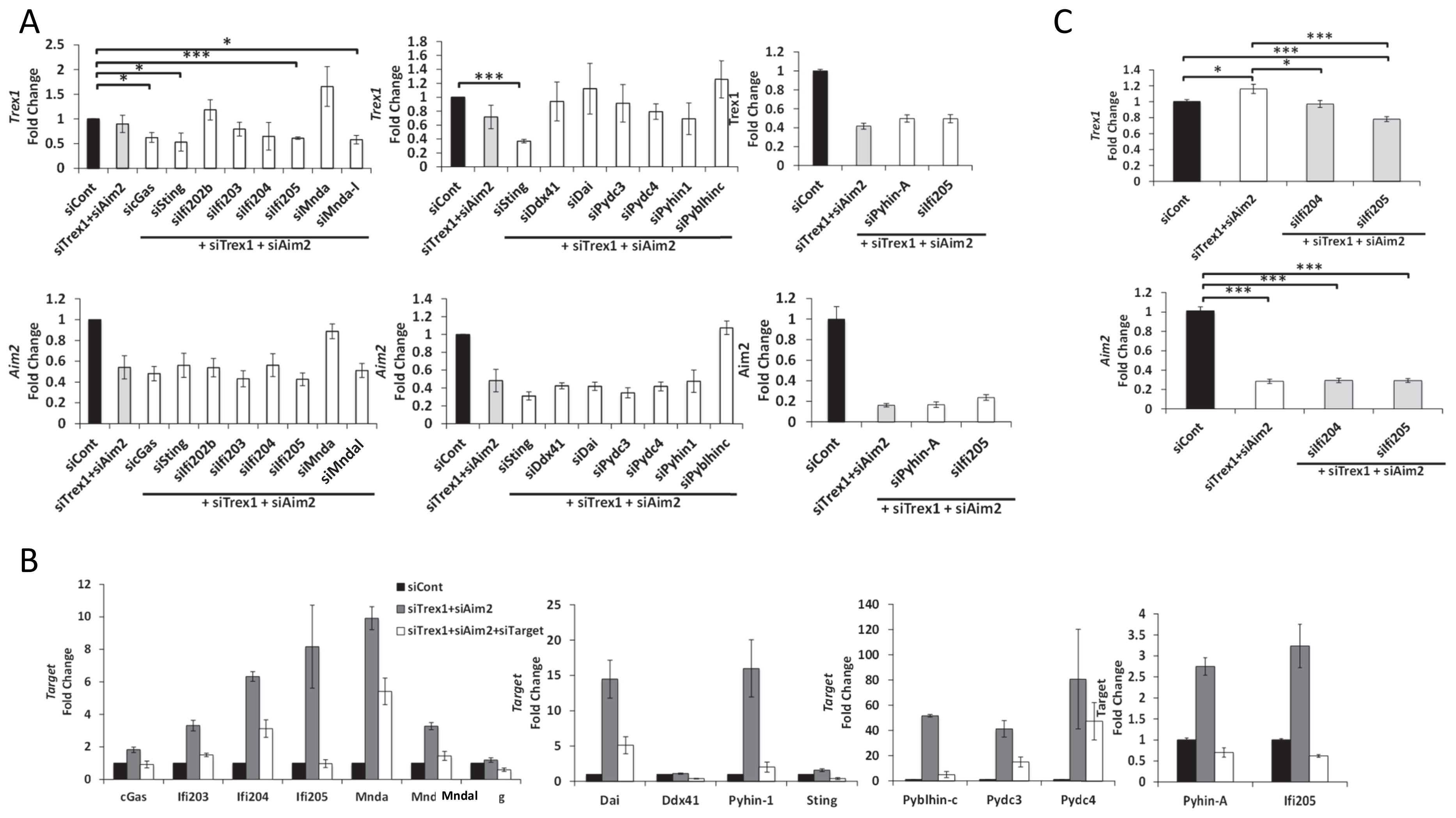

Fig. S6. Gene expression in NR9456 cells. (*A*) Trex1 and Aim2 (B) target gene expression levels were measured by RT-qPCR using same cDNAs with Fig. 4*A* to examine the knockdown effects of siRNAs. Values were normalized to *Gapdh* and are shown as mean ± SEM of three experiments in triplicate. (C) Expression levels of *Trex1* and *Aim2* were measured using same BMDC cDNAs as in Fig. 4*B,* to confirm the knockdown effects of the genes. *P<0.05 and ***P<0.0005 (two-tailed t-test)
